# Increased cytoplasmic expression of PETase enzymes in *E. coli*

**DOI:** 10.1186/s12934-024-02585-w

**Published:** 2024-11-25

**Authors:** Luke M. Carter, Chris E. MacFarlane, Samuel P. Karlock, Tridwip Sen, Joel L. Kaar, Jason A. Berberich, Jason T. Boock

**Affiliations:** 1 Department of Chemical, Paper and Biomedical Engineering, Miami University, 650 E. High St., Engineering Building 64, Oxford, OH 45056 USA; 2 Department of Chemical and Biological Engineering, University of Colorado, Boulder, CO 80309 USA

**Keywords:** PETase, Protein expression, Protein purification, SHuffle, Bioreactor

## Abstract

**Background:**

Depolymerizing polyethylene terephthalate (PET) plastics using enzymes, such as PETase, offers a sustainable chemical recycling route. To enhance degradation, many groups have sought to engineer PETase for faster catalysis on PET and elevated stability. Considerably less effort has been focused toward expressing large quantities of the enzyme, which is necessary for large-scale application and widespread use. In this work, we evaluated several *E. coli* strains for their potential to produce soluble, folded, and active *Is*PETase, and moved the production to a benchtop bioreactor. As PETase is known to require disulfide bonds to be functional, we screened several disulfide-bond promoting strains of *E. coli* to produce *Is*PETase, FAST-PETase and Hot-PETase.

**Results:**

We found expression in SHuffle T7 Express results in higher active expression of *Is*PETase compared to standard *E. coli* production strains such as BL21(DE3), reaching a purified titer of 20 mg enzyme per L of culture from shake flasks using 2xLB medium. We characterized purified *Is*PETase on 4-nitrophenyl acetate and PET microplastics, showing the enzyme produced in the disulfide-bond promoting host has high activity. Using a complex medium with glycerol and a controlled bioreactor, *Is*PETase titer reached 104 mg per L for a 46-h culture. FAST-PETase was found to be produced at similar levels in BL21(DE3) or SHuffle T7 Express, with purified production reaching 65 mg per L culture when made in BL21(DE3). Hot-PETase titers were greatest in BL21(DE3) reaching 77 mg per L culture.

**Conclusions:**

We provide protein expression methods to produce three important PETase variants. Importantly, for *Is*PETase, changing expression host, medium optimization and movement to a bioreactor resulted in a 50-fold improvement in production amount with a per cell dry weight productivity of 0.45 mg_PETase_ g_CDW_^−1^ h^−1^, which is tenfold greater than that for *K. pastoris*. We show that the benefit of using SHuffle T7 Express for expression only extends to *Is*PETase, with FAST-PETase and Hot-PETase better produced and purified from BL21(DE3), which is unexpected given the number of cysteines present. This work represents a systematic evaluation of protein expression and purification conditions for PETase variants to permit further study of these important enzymes.

**Supplementary Information:**

The online version contains supplementary material available at 10.1186/s12934-024-02585-w.

## Background

More than 370 million metric tons of plastic are produced worldwide each year with approximately 10% of it being polyethylene terephthalate (PET) [1, 2]. Plastics are versatile, inexpensive, lightweight, strong, and durable with wide use in packaging, consumer products, building and construction materials, and textiles. Many plastic products have short functional lifetimes, such as PET in water bottles and food packaging, which is often single use. The properties of plastics also make their remediation challenging due to long natural decay times, leading to estimates of 12,000 million metric tons in landfills and the environment by 2050 [2]. Creating a circular economy for plastics [1] as well as breaking down current plastic waste is paramount to avoiding such high accumulation.

Enzymatic hydrolysis of plastics has emerged over the past 20 years as a promising strategy to break down plastics into monomers that can be resynthesized into high quality plastics [3, 4]. Enzyme-based conversion is advantageous over mechanical and traditional chemical recycling as reactions take place at low temperature and in water; however, turnover is slow and often incomplete. While esterases, specifically cutinases, have shown activity on plastics, the discovery of a naturally-evolved enzyme [5], called *Is*PETase, ushered in a new wave of research due to its higher propensity to act upon PET, earning its own enzyme classification number: 3.1.1.101. While *Is*PETase is active on real PET substrates, its low catalytic rate and moderate thermostability have prompted numerous engineering efforts to improve its properties to make it more industrially useful [3]. In particular, two variants, FAST-PETase [6] and Hot-PETase [7], have become widely used. FAST-PETase combined previously engineered PETase variants with machine learning to develop an enzyme that breaks down plastic in under a week [6]. Hot-PETase resulted from numerous rounds of directed evolution and enzyme activity screening at elevated temperatures as well as rational design to produce an enzyme with substantially improved thermostability and performance on plastics [7]. Comparative performance has revealed the advantages of evolved enzymes, yet unfortunately, low conversions (< 20%) are still observed at bioreactor-scale conditions for reactions with PETases [8].

While enzyme engineering and evaluation of reactor conditions, including substrate processing and properties, have received much attention [8, 9], considerably less effort has been given to sourcing the PETase enzyme. Techno-economic analysis has found enzyme cost to account for approximately 4% of total plastic recycling cost [10, 11]; however, cost per kilogram enzyme were estimated using production values similar to that of cellulases, which have yet to be realized for recombinant enzymes such as PETase and can vary widely [12]. Additionally, scanning the literature reveals a wide array of protein production strategies for PETases, including differing organisms, strains, genetic sequences and expression strategies. Most reports which produce and study PETases do not list the enzyme titers achieved. Developing a standard production method at a moderate scale for *Is*PETase and variants serves to benefit researchers working with this class of enzymes as they move from protein engineering and characterization studies requiring small quantities of protein to process development and scale-up requiring more protein for larger reactor studies that better predict performance at an industrial scale [8].

*E. coli* remains a common expression host for recombinant proteins, including *Is*PETase and variants. Many studies use the gene for *Is*PETase encoded with its native signal peptide that can be sourced from Addgene (# 112202) combined with the *E. coli* strain C41(DE3) for expression [13]. Using this strategy and 2-YT medium, purified production amounts of 15 mg *Is*PETase per liter culture were achieved [14]. Secretion of recombinant proteins is beneficial for downstream processing and native *Is*PETase is secreted from *Ideonella sakaiensis* [5], potentially making it a strong candidate for extracellular production. Secretion from *E. coli* and *Bacillus subtilis* for *Is*PETase and variants have been explored using different signal sequences and promoters; however, most report titers below 15 mg per liter [15–18]. The most successful prokaryotic secretion method used an *aprE* signal sequence and protease-knockout strain of *B. subtilis*, finding *Is*PETase titers reaching 80 mg per liter as determined by activity in the supernatant [19]. High-density fermentations of *Komagataella pastoris* (previously *Pichia pastoris*) combined with secretion and glycosylation have proven the most successful for *Is*PETase production, reaching 1.2 g per liter as measured by total protein in the supernatant [20]. Using a similar strategy, researchers secreted FAST-PETase from *K. pastoris* using high-density fermentations and intracellular chaperones to achieve a titer of 3.23 g enzyme per liter culture [21].

Our approach to increasing PETase production amount involved screening *E. coli* expression hosts, specifically those designed to promote disulfide bond formation in the cytoplasm. PETase contains four cysteine residues, which are expected to form two disulfide bonds. The disulfide between C203 and C239 is interesting since it is evolutionarily distinct from other cutinases that have activity on PET, it is near the active site, and it has been shown to be required for activity [22, 23]. Additionally, PETase is naturally secreted from the Gram-negative *I. sakaiensis* [5], likely using the general secretory pathway to arrive in the oxidizing periplasm, which would permit disulfide bonds to form properly. RosettaGami B has an oxidizing cytoplasm due to mutations to *gor* and *trxB* (termed Origami) as well as contains a plasmid that encodes rare tRNAs and a T7 RNA polymerase (RNAP) gene under control of a *lacUV5* promoter. SHuffle has a similar set of mutations to *gor* and *trxB* to promote disulfide bond formation as well as a cytoplasmic copy of the chaperone disulfide bond isomerase (*dsbC*), which is naturally used to rearrange misconnected disulfides [24]. Similar to RosettaGami B, SHuffle T7 Express has a T7 RNAP gene to permit use with ubiquitous pET plasmid series for high level expression; however, the T7 RNAP gene has been inserted in place of *lacZ* and as such is under the control of the *lac* promoter. Others have used Origami or SHuffle T7 Express for production of *Is*PETase [25] and variants, but few report amounts produced. A mutant PETase was produced with chaperones and autoinduction media in SHuffle T7 Express at 80 mg per liter as measured by activity in lysate. Hot-PETase was found to be made and purified at 12 mg per liter and 110 mg per liter when produced from SHuffle T7 Express [9] and Origami II [7] cells, respectively.

To better understand the impact of a reducing or oxidizing cytoplasm, we measured the soluble production of *Is*PETase and variants FAST-PETase and Hot-PETase in several *E. coli* strains. Comparisons were made to other strategies that have been used to make PETase in *E. coli*. Media optimization and culturing in a controlled benchtop bioreactor was performed for *Is*PETase to further elevate production. Few studies have moved SHuffle T7 Express to even the benchtop bioreactor scale [26–29], representing an important step to utilizing this strain that has shown promise in making proteins with multiple disulfides. We also provide characterization of the enzymes using the soluble, colorimetric esterase substrate 4-nitrophenyl acetate (4-NPA) as well as PET microparticles to show the enzymes produced using our methods have similar activity to those made elsewhere. Taken together, this study represents an important standardization for the soluble production of PETase enzymes using common *E. coli* strains, which benefits both laboratory researchers and those working to transition enzymatic recycling to an industrial scale.

## Methods

### Plasmid construction and strains

The gene encoding PETase from *Ideonella sakaiensis* (*Is*PETase) was codon optimized for expression in *E. coli* without its 26 base pair signal sequence as was done previously [5] (Supplemental Information). The gene was added to a pET21b or pET24a plasmid between *ndeI* and *xhoI* cutsites, which appends the protein with a C-terminal hexa-histidine tag. Traditional restriction enzyme-based cloning followed by ligation were used to generate all plasmids (all supplies from NEB). Assembled plasmids were transformed into DH5α chemically competent *E. coli* cells for plasmid amplification and cryogenic storage at -80 ºC in 15% (v/v) glycerol. After plasmid DNA isolation (Zymo), Sanger or whole-plasmid sequencing was performed to verify assembly (Azenta). Genes encoding FAST-PETase (S121E, D186H, R224Q, N233K, R280A) [6] and Hot-PETase (S58A, S61V, R90T, K95N, Q119K, S121E, M154G, P181V, Q182M, D186H, S207R, N212K, S213E, S214Y, R224L, N233C, N241C, K252M, T270Q, R280A, S282C) [7] were synthesized as gBlocks (IDT) with identical codons as wild-type *Is*PETase used in this study, except internal *ncoI*, *bsaI*, and *sphI* sites were removed with synonymous codons (Supplemental Information). Note, all numbering for PETase amino acids used in this paper is based on the primary sequence with the signal peptide as this is what is commonly used in the literature; to determine the amino acid number for the proteins in this study, subtract 26. Genes were added between *ndeI* and *xhoI* sites of pET21b. Select plasmids from this study are available upon request from Addgene. Additionally, pET21b( +)-Is-PETase with a signal sequence was purchased from Addgene (# 112202) and pGro7 from Takara.

After sequence verification, isolated plasmids were transformed into chemically competent BL21(DE3) (Novagen), Rosetta Gami B(DE3) (Novagen), Shuffle T7 Express (NEB), C41(DE3) (Lucigen), or C43(DE3) (Lucigen), and were plated on Luria Bertani (LB) agar containing 100 µg mL^−1^ ampicillin (pET21b) or 50 µg mL^−1^ kanamycin (pET24a). Strains with pGro7 were also grown in the presence of 34 µg mL^−1^ chloramphenicol. Cultures from colonies were grown at 37 ºC at 250 RPM in LB medium with the same concentration of antibiotics prior to cryostorage in 15% (v/v) glycerol at -80 ºC.

### Medium preparation and protein expression

2xLB medium contained: 10 g L^−1^ yeast extract, 20 g L^−1^ tryptone, and 5 g L^−1^ NaCl. TB medium (Difico) was prepared according to manufacturer directions. Defined medium [30] contained:13.3 g L^−1^ KH_2_PO_4_, 4 g L^−1^ (NH_4_)_2_HPO4•H_2_O, 1.7 g L^−1^ citrate, 1.2 g L^−1^ MgSO_4_•7H_2_O, 4.5 mg L^−1^ thiamine HCl, and 1 × trace metals mix. The 100 × trace metals mix contained: 10 mg L^−1^ H_3_BO_3_, 10 mg L^−1^ CoCl_2_•6H_2_O, 2.5 g L^−1^ ZnSO_4_•7H_2_O, 400 mg L^−1^ MnCl_2_•4H_2_O, 10 mg L^−1^ Na_2_MoO_4_•2H_2_O, 180 mg L^−1^ CuSO_4_•5H_2_O, 2 g L^−1^ FeSO_4_•7H_2_O, 10 mg L^−1^ NiSO_4_•6H_2_O. Semi-defined medium [30] contained: 13 g L^−1^ KH_2_PO_4_, 10 g L^−1^ K_2_HPO_4_, 4.6 g L^−1^ NaH_2_PO_4_, 3 g L^−1^ (NH_4_)_2_HPO4•H_2_O, 2 g L^−1^ MgSO_4_•7H_2_O, 73.8 mg L^−1^ CaCl_2_•2H_2_O, and 1 × trace metals mix. Complex medium [30] contained: 5 g L^−1^ yeast extract, 10 g L^−1^ tryptone, 10 g L^−1^ NaCl, 8.34 g L^−1^ KH_2_PO_4_, 6.74 g L^−1^, 0.5 g L^−1^ MgSO_4_•7H_2_O, 50 mg L^−1^ CaCl_2_•2H_2_O, and 1 × trace metals mix. Non-defined components with base salts as well as 580 g L^−1^ glycerol were autoclaved prior to use. Stock 1 M MgSO_4_•7H_2_O, 1 M CaCl_2_•2H_2_O, 1 M isopropyl ß-D-1-thiogalactopyranoside (IPTG), 1 g mL^−1^ thiamine HCl, 200 g L^−1^ glucose, 200 g L^−1^ L-arabinose, and 100 × trace metals mix were sterile filtered (0.2 µm) prior to use. Defined, semi-defined, and complex medias were prepared fresh prior to use.

Overnight cultures were started in identical medium as was used for expression using cells from freezer stocks. Final antibiotic concentration were 100 µg mL^−1^ ampicillin (pET21b), 50 µg mL^−1^ kanamycin (pET24a), or 34 µg mL^−1^ chloramphenicol (pGro7). Overnight cultures were grown at 37 ºC at 250 RPM for 16 to 20 h. Optical density at 600 nm (OD_600_) (Genesys) was measured. Subcultures were normalized to an optical density of 0.05 A.U.. Small-scale expression experiments were carried out in 25 mL of medium in 125 mL baffled shake flasks (Kimble KIMAX). Cell growth occurred at 37 ºC at 250 RPM in a shaker incubator (New Brunswick Innova 42) until an OD_600_ of 0.6 A.U. to 0.8 A.U. was reached. At that time, protein production was initiated by the addition of IPTG to 0.5 mM final concentration. For cells with pGro7, chaperone expression was induced for with 5 g L^−1^ arabinose at the same time IPTG was added. After inducers were added, incubator temperature was dropped to 20 ºC and protein expression was carried out for 24 h unless otherwise noted. An OD_600_ reading was taken prior to centrifuging 5 mL or 10 mL of culture at 4000 rcf for 10 min. The supernatant was removed and cell pellet stored at -80 ºC prior to further analysis.

### Cell lysis and active PETase quantification

Frozen cell pellets were defrosted at room temperature prior to resuspension in 20 mM sodium phosphate buffer (pH 7.4). For lysis per unit volume, 50 µL volume of buffer was added per 1 mL of culture harvested. For lysis per unit optical density, cells were resuspended in buffer to achieve an OD_600_ of 100 A.U.. Resuspended pellets were transferred to a 1.5 mL centrifuge tube, and lysozyme was added to 1 mg mL^−1^ and Triton 100 × was added to 1μL mL^−1^. Lysis occurred on a multi-tube rocker-rotator (VWR) set to 15 rpm for 20 min at room temperature. After initial lysis, DNA disruption was achieved by addition of DNase (Invitrogen) to 5 U mL^−1^ total lysis volume. Tubes were rotated for an additional 20 min at room temperature. Crude lysate-containing tubes were centrifuged for 30 min at 4 °C and 22,000 rcf, and the supernatant was retained as clarified lysate.

PETase activity was measured in soluble clarified lysates by activity on 4-nitrophenyl acetate (4-NPA) (Sigma). Samples were set up in a clear 96-well plate with each well containing 100 μL total volume with: 80 μL of 100 mM potassium phosphate buffer (pH 7), 10 μL of lysate, and 10 μL of 10 mM 4-NPA. Stock 10 mM 4-NPA was prepared in acetonitrile. Lysates were diluted over a wide range using 100 mM potassium phosphate buffer (pH 7), targeting a diluted activity of ~ 0.025 to 0.05 units per well. A unit (U) is defined as 1 μmol 4-NPA turnover to 4-nitrophenol per minute. The extinction coefficient for 4-nitrophenol was determined by dilution into 100 mM potassium phosphate buffer (pH 7) (Fig. S1) and was found to be 11,627.5 M^−1^ cm^−1^ (assumed pathlength of 2 mm). Controls without PETase (10 μL additional buffer) were similarly set up to subtract off background rate due to non-enzymatic hydrolysis of 4-NPA. Absorbance at a wavelength of 405 nm was measured on a microplate reader (Biotek) every 15 s for 10 min at 25 °C. Active PETase concentration, expressed as units of PETase per liter of total culture, was determined by analysis of the initial rate of absorbance change over time, subtraction of a blank rate, and multiplication by appropriate dilution factors. Absorbances below 0.8 A.U. (~ 300 µM product) were used for initial rate calculations. Samples with rates above 0.14 A.U. min^−1^ (6 mU min^−1^) were eliminated and further diluted to ensure sufficient data points to determine a catalytic rate and that the rate was linear with PETase concentration. Additionally, initial rate was divided by total protein concentration and appropriate dilution factors to calculate units of PETase per gram of total protein. Statistical analysis used a two-tailed Student’s t-test assuming equal variance.

### Total protein quantification

Total protein quantification was carried out using a Pierce BCA kit according to manufacturer specifications and absorbance quantified using a microplate reader (Biotek) at 562 nm. For each set of buffers used, a buffer blank was included and the resulting absorbance was subtracted from that of the sample. Total protein amount was determined through comparison to bovine serum albumin standards (0.025 mg mL^−1^ to 2.0 mg mL^−1^) with buffer blank subtracted. A linear fit equation through all standard absorbances with a set intercept of 0 A.U. was used to quantify total protein concentration.

### Protein purification

For quantitative protein purification, PETase expression was scaled up to 500 mL culture in a 2-L baffled flask (Pyrex). All other protein production steps were identical to those used for small-scale experiments. Cell rupture occurred as previously described where the amount of lysis buffer was scaled by final culture optical density, resulting in an OD_600_ of 100 A.U.. An additional sonication step was performed after the DNase treatment of 30% duty cycle for 1.5 min on ice (3x). To clarify lysates, samples were centrifuged at 18,500 rcf for 30 min at 4 °C. Samples were filtered through a 0.2 µm filter prior to purification.

Proteins were purified with an AKTA Go FPLC using two chromatography steps: immobilized metal affinity chromatography (IMAC) followed by desalting into storage buffer. For IMAC, the binding buffer (A) was 20 mM sodium phosphate buffer (pH 7.4) with 500 mM NaCl and 20 mM imidazole, and elution buffer (B) was 20 mM sodium phosphate (pH 7.4), 500 mM NaCl, 500 mM imidazole. Imidazole and NaCl were added to clarified lysates to final concentrations of 20 mM and 500 mM, respectively, to eliminate non-specific interactions. A 1 mL HisTrap HP (Cytiva) column was used with a flow rate of 1 mL min^−1^, unless otherwise noted. After clarified lysate was applied to the column, the column was washed with 100% A buffer until absorbance readings were lower than 100 mA.U.. A single step elution, unless otherwise noted, was carried out using 50% B buffer (250 mM imidazole) and 0.5 mL fractions collected. Activity on 4-NPA was used to identify high activity fractions. Proteins were desalted into 50 mM sodium phosphate (pH 7), 30 mM NaCl, 0.01% (w/v) sodium azide using a 5 mL HiTrap PD-10 column (Cytiva). Purified samples were stored at 4 °C until use.

Total protein concentration was measured using BCA and comparison to BSA standard curve, as described above. Protein purity was assessed by SDS-PAGE (all supplies from BioRAD). Samples were denatured in 1 × Laemmli buffer with β-mercaptoethanol at 95 °C for 10 min. Samples containing 2.5 µg of total protein were loaded on a Stain-free Any kDa gel and separation occurred for 35 min at 150 V using 1 × tris/glycine/SDS buffer. Precision plus unstained molecular weight standards were used to assess molecular weight and semi-quantify amount. To visualize proteins, Stain-free activation occurred for 5 min followed by visualization using a Gel Doc XR + and gel analysis used Image Lab.

### Specific activity and Michaelis–Menten constant determination

Specific activity, *k*_*cat*_, and *K*_*M*_ were determined for PETase and variants using 4-NPA at 25 °C, similar to [31, 32]. Samples were set up in a clear 96-well plate with each well containing 100 μL total volume with: 80 μL of 100 mM potassium phosphate buffer (pH 7), 10 μL of sample, and 10 μL of 4-NPA. Controls without PETase (10 μL additional buffer) were similarly set up to subtract off background rate due to non-enzymatic hydrolysis of 4-NPA. After initial mixing, absorbance at a wavelength of 405 nm was measured on a microplate reader (Biotek) every 15 s for 10 min. All characterized proteins were produced in SHuffle T7 Express.

For specific activity, the final concentration of 4-NPA was 1 mM and various PETase concentrations were evaluated (0.5 μg mL^−1^ to 20 μg mL^−1^ final concentration), each in triplicate. Initial rate was determined until an absorbance of 0.8 A.U. was reached. Samples with activity similar to background buffer or which reached an absorbance of 0.8 A.U. before 5 min were eliminated. A unit (U) is defined as 1 μmol 4-NPA turnover to 4-nitrophenol per minute. Subtracted units were plotted against total protein concentration to determine the specific activity by fitting a linear line through all data and setting the y-intercept to 0 (Kaleidagraph). To determine *k*_*cat*_ and *K*_*M*_, samples contained approximately 1 μM PETase (actual concentrations listed for each variant). 4-NPA concentrations were tested between 1 and 20 mM, each in triplicate. Background hydrolysis was measured at each 4-NPA concentration to be subtracted from the initial rate with enzyme. Subtracted initial rates were plotted as a function of 4-NPA concentration and fit to the Michaelis–Menten equation (Kaleidagraph).

### Microparticle assay

PETase catalysis of insoluble PET microparticles (Goodfellow, ES30-PD-000131, < 300 µm, 40% crystallinity) was performed as proof of activity on real substrates using a method adapted from [9]. Samples were prepared in 15-mL polypropylene tubes (Falcon), with each containing 5 mL of sodium phosphate buffer (pH 9), 150 mg PET particles (final concentration 30 mg mL^−1^), and PETase at a desired ratio to PET particles (0.34 mg_PETase_ g_PET_^−1^, 0.69 mg_PETase_ g_PET_^−1^, 1 mg_PETase_ g_PET_^−1^). Blank samples contained no PETase. HiC (Strem) concentration was determined by BCA to be 28 mg mL^−1^ and was assumed pure after analysis by SDS-PAGE. Reactions were carried out in an Eppendorf ThermoMixer C at 1000 rpm and 37 °C. At each timepoint, 500 μL of sample was removed and centrifuged at 13,000 rcf for 5 min to pellet insoluble remaining PET. The supernatant was removed and reaction was quenched using 2 N HCl at a ratio of 10 μL HCl to 200 μL of sample supernatant. Samples with high concentration of products were cloudy, requiring acetonitrile addition to become clear (up to two-times the final volume). Samples were stored at -20 °C prior to analysis by HPLC.

### High performance liquid chromatography

Standards of terephthalic acid (TPA) (Sigma), 2-hydroxyethyl terephthalic acid (MHET), and bis(2-hydroxyethyl) terephthalic acid (BHET) (Sigma) were prepared in order to quantify reaction products from microparticle catalysis. TPA was dissolved in 20 mM sodium phosphate buffer (pH 7.4) to a concentration of 2 mM. The pH of the solution was adjusted to 7 by dropwise addition of 1.25 N NaOH until the solution became clear and volumetric flask used to achieve the final desired volume. BHET was dissolved to a concentration of 2 mM in 24% (v/v) acetonitrile and 76% (v/v) 100 mM potassium phosphate (pH 7) buffer as BHET has poor solubility in water [33]. Further dilution of the 2 mM BHET solution used the 24% acetonitrile-buffer solvent to maintain BHET solubility.

MHET was not commercially available, requiring production by enzymatic hydrolysis of BHET using *Humicola insolens* cutinase (HiC, product Novozym® 51,032). BHET was dissolved in 24% (v/v) acetonitrile and 76% (v/v) 100 mM potassium phosphate (pH 7) buffer before addition of 0.1 g_HiC_ g_BHET_^−1^. The reaction volume was 30 mL. Reactions were carried out at 40°C and mixed at 600 rpm using a Chemglass heated magnetic mixer. A negative control reaction without HiC addition was included in the experimental setup. Samples of reaction contents were drawn at 0, 3, 24, 48, and 72 h to determine the extent of reaction from BHET to MHET as measured by HPLC peak area. The reaction was quenched by addition of 50 µL 2 N HCl per 1 mL sample. After 77 h, an additional 0.1 g_HiC_ g_BHET_^−1^ was added to complete the reaction, which took 144 total hours. The purest MHET sample was determined to be 1.89 mM by HPLC analysis comparing peak areas of BHET to MHET, representing 94% percent conversion. Further dilution of the 1.89 mM MHET solution to desired concentrations used the 24% acetonitrile-buffer solvent.

Soluble aromatic products TPA, MHET, and BHET produced from reaction of PETase on PET microparticles were separated using high performance liquid chromatography (HPLC). Reverse phase chromatography was performed using Agilent Polaris 3 C18-A column (150 × 3.0 mm, 3 µm) and Thermo HPLC system. Solvents were: (A) water with 0.1% (v/v) formic acid and (B) acetonitrile with 0.1% (v/v) formic acid. Equilibration occurred with 5% B at 0.5 mL min^−1^ and the column oven was set to 60°C. Sample injections were 5 µL. Gradient profile was adapted from [34], consisting of: 5% B for 2 min, 5% B to 17% B from 2 to 4 min, 17% B to 25% B from 4 to 6 min, 25% B to 40% B from 6 to 10 min, hold at 40% B from 10 to 12 min and 5% B re-equilibration from 12 to 16 min. Retention times, peak separation, and peak area to concentration correlations for TPA, MHET, and BHET were determined for range of concentrations from 0.05 to 1 mM (Fig. S2). Linear regression with a set y-intercept of 0 was used to determine an equation for converting peak area to analyte concentration.

### Size exclusion chromatography

Size exclusion chromatography (SEC) was used to assess the oligomerization state of PETase. An AKTA Go outfitted with a Superdex 75 Increase 10/300 GL column (Cytiva) was used for the separation. The method was isocratic using 50 mM sodium phosphate (pH 7), 30 mM NaCl, 0.01% (w/v) sodium azide flowing at 1 mL min^−1^. UV absorbance was measured to determine protein retention times. A 100 µL sample of purified PETase was separated and 0.5 mL fractions were collected for further analysis of esterase activity on 4-NPA. Only *Is*PETase was analyzed and it was purified from SHuffle T7 Express cultures. Blue dextran (Promega) was used to determine the void volume and protein standards (Sigma) were used to create a standard curve to determine molecular weight.

### Circular dichroism

Far UV circular dichroism (CD) scans of purified PETase proteins were used to determine secondary structure. PETase samples were made to 0.03 mg mL^−1^ in 10 mM sodium phosphate buffer (pH 8) and placed in a clean 1 cm quartz cuvette (Fisher Scientific). Scans between 195 and 260 nm were made using an Aviv Biomedical Model 435 circular dichroism spectrophotometer. Wavelength step size was 1 nm and three scans were completed per sample. Scans were averaged and a buffer blank subtracted prior to calculating mean residue ellipticity [35].

### Bioreactor

Bioreactor trials were carried out in an Eppendorf 2-L benchtop bioreactor connected to BioFlo 120 control unit. A 25 mL starter culture in a 125 mL baffled flask (Kimble KIMEX) was inoculated from a freezer stock and grown at 37 °C and 250 rpm for 16 to 20 h. Starter cultures were made in identical media to that used the bioreactor. A volume of the overnight culture was added to the 1.5 L of medium in the reactor to achieve an OD_600_ of 0.05 A.U.. Cells were grown at 37 °C until an OD_600_ between 0.6 A.U. and 0.8 A.U. was reached, at which time protein production was induced with 0.5 mM IPTG and the reactor temperature was dropped to 20 °C. Agitation speed was initially set to 300 rpm and air flow rate to 1.5 L min^−1^. Cascade control was used to keep the dissolved oxygen amount greater than 30% of saturation by varying impeller speed (max 800 rpm) and air flow rate (max 15 L min^−1^). A pH window was set between 6.8 and 7.2 using addition of either ammonium hydroxide or hydrochloric acid. In addition to the bioreactor, a 100 mL shake flask culture in a 500 mL baffled flask (Kimble KIMEX) was grown using similar setpoints (temperature and induction) as the bioreactor to compare scales and impact of control of process variables. The culture in the shake flask did not have pH or dissolved oxygen control, and was mixed at 250 rpm in a shaker incubator (New Brunswick Innova 42).

At each timepoint following induction, 1.5 mL samples were removed for analysis. After measuring OD_600_, samples were centrifuged at 20,000 rcf for 5 min at room temperature. The supernatant was removed to determine glycerol substrate consumption and acetate product buildup. Glycerol and acetate were separated using HPLC and an Aminex HPX-87H column (BioRad) at 30 °C with isocratic 0.5 mM H_2_SO_4_ flowing at 0.6 mL min^−1^ [36]. Compounds were quantified using refractive index and comparisons to standards of pure glycerol and acetate at known concentrations. Culture pellets were stored at -80 °C for activity analysis in lysate as described above.

A second bioreactor run was carried out for complex medium with 35 g L^−1^ glycerol to determine reproducibility as well as harvest culture for purification. Time to harvest was determined by taking samples once per hour to track the accumulation of acetate and PETase activity to determine when the peak activity occurred. Once a measurement was made that had decreased activity, the final volume of the reactor was measured (1.3 L) and split into two centrifuge bottles to harvest. The cell pellet from one of the bottles was lysed and *Is*PETase purified using identical procedures as above except a 5 mL HisTrap HP column and 53 mL HiPrep 26/10 Desalting column were used due to the larger scale. Final protein concentration was determined using BCA and purity assessed by SDS-PAGE, both described above.

## Results:

### Screening IsPETase production hosts

Production using a codon-optimized *Is*PETase without a signal sequence and with a C-terminal hexa-histidine tag (Supporting Information) was initially carried out in BL21(DE3) as it is a common laboratory protein production strain. The medium used was 2xLB and protein expression was induced with 0.5 mM IPTG for 24 h at 20 °C as similar protocols have proven successful for *Is*PETase in the past [8]. We found approximately 14 units (U, μmol_product_ min^−1^) of *Is*PETase produced per liter of culture volume as measured on the soluble substrate 4-nitrophenol acetate (4-NPA) (Table S1). A control was performed where an empty pET21b plasmid was used in place of pET21b *Is*PETase, finding 2.6 units per liter culture, suggesting that *Is*PETase is in fact being made. We screened several parameters including induction temperature (37 °C), inducer concentration (0.05 mM IPTG), and expression time (48 h), none of which led to large improvements in soluble and active enzyme production, and in the case of raising the induction temperature, significantly less enzyme activity (Table S1).

**Fig. 1:**
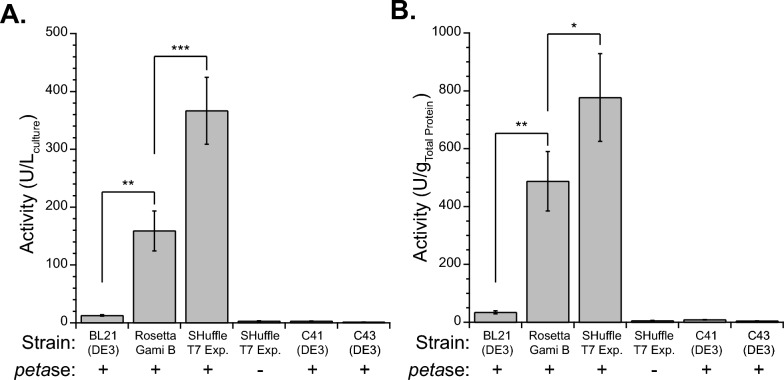
*Is*PETase production in various strains of *E. coli*. Production of *Is*PETase as measured by enzyme activity on 4-NPA in clarified cell lysates. Lysis amount was based on culture volume. Activity was normalized to **A** culture volume and **B** total protein. Activity unit (U) is defined as 1 μmol min^−1^ product formed using 1 mM 4-NPA at room temperature in 100 mM potassium phosphate buffer (pH 7). Strains contained pET21b PETase ( +) or pET21b Empty (–). Average of at least 3 independent cultures shown and error bars represent the standard deviation. Shown are selected Student’s t-test results, * (p < 0.05), ** (p < 0.005), *** (p < 0.0005); statistical comparisons between all samples can be found in the Supplementary Data File

We hypothesized the limitation we observed in production of *Is*PETase in BL21(DE3) was due to the lack of disulfide bond formation in the reducing cytoplasm, and thus potentially *Is*PETase instability. We evaluated two common cytoplasmic disulfide bond forming *E. coli* hosts, RosettaGami B(DE3) and SHuffle T7 Express, for their ability to produce *Is*PETase. Using the same expression conditions as before, *Is*PETase production was enhanced when produced in RosettaGami B and SHuffle T7 Express, reaching 159 ± 34 and 367 ± 58 units per liter culture, respectively (Fig. 1a). To ensure that the SHuffle T7 Express was not causing the activity difference, an empty pET21b plasmid was added to SHuffle T7 Express. The background activity of lysates without *Is*PETase on 4-NPA was 2.8 ± 0.6 units per liter culture. These results suggest a vast improvement of cytoplasmic *Is*PETase production when a disulfide bond forming host was used and further enhancement by including a cytoplasmic copy of *dsbC*. We also measured the total protein present in each lysate sample in order to normalize activity to total protein to control for growth and lysis differences finding similar improvement (Fig. 1b). We attempted to further increase *Is*PETase production by utilizing a different plasmid, pET24a, as well as co-expressing with chaperones GroEL/ES, finding neither resulted in more *Is*PETase production (Supplementary Note 1, Table S2).

Cell growth was monitored by optical density to ensure differences in production due to the alternate strains were not due to growth. The SHuffle T7 Express cells grew to a lower optical density of 4.0 ± 0.3 A.U. compared to 6.8 ± 0.2 A.U. for BL21(DE3). Rosetta-Gami B(DE3) grew to an even lower optical density of 1.7 ± 0.1 A.U.. The BL21(DE3) cultures consistently grew to almost double the optical density of the SHuffle T7 Express cultures as measured at 24 h post induction. To ensure that the cell rupture step was not limiting, BL21(DE3) and SHuffle T7 Express samples were also lysed with the volume of lysis buffer scaled by optical density. When analyzing active protein using this method, the SHuffle T7 Express samples were found to have 493 ± 55 units per liter culture and BL21(DE3) had 37 ± 4 units per liter culture (Table S2). When normalizing by total protein, BL21(DE3) had 47 ± 10. units per gram, and SHuffle T7 Express had 603 ± 3 units per gram. Using results from the optical density normalized lysis, SHuffle T7 Express produced approximately 13-fold more active *Is*PETase than BL21(DE3).

We next sought to compare the SHuffle T7 Express host to other *E. coli* strains that are commonly used to produce *Is*PETase, mainly C41(DE3) [13]. C41 and C43 OverExpress strains have genetic mutations that enhance the production of toxic proteins. Using the plasmid assembled for this study, we found the C41(DE3) and C43(DE3) strains to produce 2.9 and 1.3 units per liter culture, much lower than that of BL21(DE3) and similar to that of cells without *petase* (Fig. 1a). Surprised by this result, we screened 8 C41(DE3) colonies to ensure all had low activity, which was verified with the highest activity being 3.1 units per liter culture (Table S3). Compared to the commonly used plasmid for *Is*PETase production from the plasmid repository Addgene, the *petase*_*Is*_ gene used in this study has different codon optimization and does not contain a signal sequence. The Addgene plasmid was purchased and tested in C41(DE3) and SHuffle T7 Express, finding the C41(DE3) cells produced 107 units per liter culture and SHuffle T7 Express only made 10.0 units per liter culture, both significantly different than when the signal sequence was removed (Table S4). This is unsurprising given the ability of signal sequences to be utilized across species and the benefit to using SHuffle is tied to cytoplasmic expression. We did not measure activity in the supernatant to see if the enzyme was secreted outside the cells. Although commonly used C41(DE3) did have much more intracellular expression when a version of *petase*_*Is*_ with a signal sequence was produced, it was still 3.4-fold lower than when SHuffle T7 Express was used with a construct that does not contain a signal sequence.

### Purification and characterization of IsPETase

*Is*PETase was purified to determine the amount of protein produced (Table 1). Purification from BL21(DE3) resulted in 1.6 mg of protein at a purity of approximately 50 to 65% as measured by densitometry (Fig. S3). The lack of purity from BL21(DE3) is not surprising given its low production and is likely due to non-specific binding during the IMAC step. Production from SHuffle T7 Express was 10.5 mg from a 500 mL culture at an estimated 99% purity. Taken together, the purification results suggest a 13-fold improvement upon use of the disulfide bond promoting host, identical to the values found when comparing activities in lysate. Few reports list the purified titer of the original *Is*PETase, with one finding 15 mg of purified protein per L culture when produced from C41(DE3) [14]. Here, we were able to produce 21 mg per L, which represents a 25% improvement. Circular dichroism confirmed that the soluble protein was well-folded (Fig. S4). Size exclusion chromatography showed that *Is*PETase is monomeric, as has been found previously (Fig. S5) [23]. Further, activity on 4-NPA was measured for SEC fractions (Fig. S5), confirming the single peak has all the esterase activity.

**Table 1:** *Is*PETase Purification

	Step	Production amount (mg/L_culture_)^a^	Amount Yield^b^	Purity^c^
BL21(DE3)	Lysate	2.34 ± 0.16	–	–
	IMAC	2.25 ± 0.01	0.96 ± 0.06^d^	0.52
	Desalted	2.17 ± 0.02	0.97 ± 0.01	0.66
SHuffle T7 Express	Lysate	82.8 ± 5.5	–	–
	IMAC	21.7 ± 0.5	0.26 ± 0.02	0.99
	Desalted	21.0 ± 0.5	0.96 ± 0.03	0.99

^a^Amount of *Is*PETase after purification from 0.5 L of culture. Amount for the IMAC and desalted steps were measured by BCA and comparison to BSA standard. Amount for lysate step was estimated using the 4-NPA activity and specific activity (see Fig. S6). Error represents standard deviation of three replicate assay samples. Amount was scaled to 1 L culture volume

^b^Amount yield represents the retention of amount in each purification step and error is propagated

^c^Purity is estimated from gel analysis of Fig. S3

^d^Amount yield for BL21(DE3) is overestimated due to the purity of the IMAC step only being approximately 50% pure and total protein concentration being used for this calculation

To ensure the *Is*PETase produced from SHuffle had similar activity to the enzyme produced in other hosts, catalysis was measured on soluble 4-NPA and insoluble PET microparticle substrates. Initial characterization was performed using 1 mM of 4-NPA at a variety of PETase concentrations to determine the specific activity for the enzyme as well as ensure that activity measurements are linearly dependent on protein concentration. Using these conditions, we found that PETase had a specific activity of 5.26 ± 0.14 U/mg or 0.151 ± 0.035 U/nmol (Fig. S6), with a linear dependence. Michaelis–Menten parameters were determined for 4-NPA (Fig. S7), providing values of 8.16 ± 0.66 mM for *K*_*m*_ and 30.3 ± 1.1 s^−1^ for *k*_*cat*_. These values compare similarly to the literature values of *K*_*m*_ of 4.6 mM and *k*_*cat*_ of 27 s^−1^ for wild-type PETase on 4-NPA, albeit their characterization was done at 30 °C and ours at 25 °C, both in pH 7 buffer [31].

**Fig. 2:**
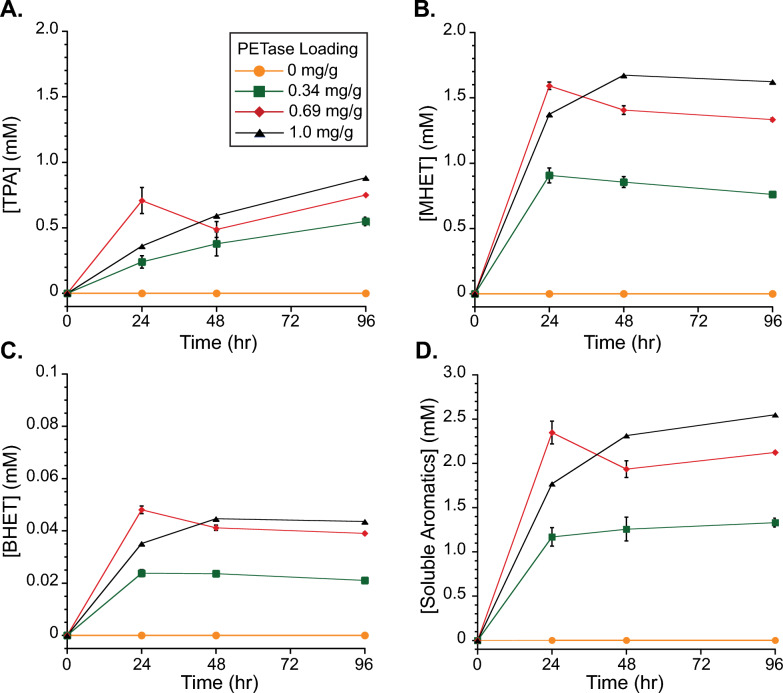
*Is*PETase Activity on PET microparticles. *Is*PETase activity was measured by accumulation of soluble aromatic products over time: **A** TPA, **B** MHET, **C** BHET, and **D** total soluble aromatics. Individual series in each plot represent PETase loadings of 0 (orange circles, n = 2), 0.34 (green squares, n = 2), 0.69 (red diamonds, n = 2), and 1.0 (black triangles, n = 1) milligrams of enzyme per gram of PET (mg g^−1^). Reactions occurred at 37 °C in 50 mM sodium phosphate buffer (pH 9), contained 30 mg PET per mL reaction, and were mixed at 1000 rpm using an Eppendorf thermomixer. Error bars represent the standard deviation of samples

*Is*PETase activity was determined using PET microparticles to represent realistic substrates. Samples were prepared with three ratios of *Is*PETase to PET particles: 0.34, 0.69, and 1.0 mg_PETase_/g_PET_ and soluble TPA, MHET and BHET were measured over time (Fig. 2). After 96 h, reactions containing 0.34 mg g^−1^, 0.69 mg g^−1^, or 1 mg g^−1^ *Is*PETase released approximately 1.3, 2.1, and 2.5 mM product, over a thousand times higher than the sample without *Is*PETase. For 1 mg g^−1^ at 48 h, 1.76 mM of soluble aromatic products were formed, which compares quite similarly to 1.72 mM released when measured in a previous study using similar reaction conditions (40 °C, 29 mg mL^−1^ PET loading, 1 mg *Is*PETase per gram PET) [9]. These results suggest that the *Is*PETase produced in this study without a signal sequence and in a disulfide-bond promoting host has similar activity as those produced using other methods.

### Medium optimization for IsPETase production

Given the success of SHuffle T7 Express to increase the production of *Is*PETase, we next performed a medium optimization. While using a non-defined, complex medium such as 2xLB is possible, moving to a more defined medium with additional carbon and nitrogen sources could benefit both economics and expression [37]. As such, six different medias were assessed at 25 mL scale for active *Is*PETase production with varying carbon sources and inducer molecules (Table S5). For defined [38] and semi-defined [39] medias, recipes were selected that have previously shown success in increasing biomass and recombinant protein production in *E. coli* [30]. Three non-defined medias were tested, 2xLB, TB, and complex medium [30, 40].

Comparing the *Is*PETase activity in 2xLB and Terrific Broth (TB), more than double the activity was observed with 2xLB despite cells growing to double the optical density in TB (Table 2). When defined, semi-defined or complex media was tested with glucose as a carbon source, cells all reached higher optical densities than 2xLB, but the best producing complex medium sample only had half of the activity of 2xLB (Table 2). Due to the placement of the T7 RNAP gene in the *lac* operon in SHuffle [24], production of T7 RNAP is catabolite repressed resulting only in expression of desired protein when glucose levels are depleted, providing a reason why *Is*PETase levels were low even after complete consumption of glucose. Additionally, the genetic construction of SHuffle does not permit lactose to be used as an inducer due to the knockout of *lacZ* [24]. Given the partial success of the complex medium, 35 g L^−1^ glycerol was substituted for glucose as a carbon source. Using this medium in 25-mL batch cultures, production reached 980 U per L culture with an optical density of almost 10 A.U., both improvements over 2xLB.

**Table 2:** Medium optimization for *Is*PETase production in shake flasks

	2xLB	TB	2xLB Glucose	Defined^a^	Semi-defined^a^	Complex^a^ Glucose IPTG	Complex^a^ Glycerol IPTG
Carbon source	–	–	5 g/L glucose	5 g/L glucose	5 g/L glucose	5 g/L glucose	35 g/L glycerol
Inducer	0.5 mM IPTG	0.5 mM IPTG	0.5 mM IPTG	0.5 mM IPTG	0.5 mM IPTG	0.5 mM IPTG	0.5 mM IPTG
Induction OD_600nm_ (A.U.)	0.76 ± 0.09	0.83 ± 0.03	0.85 ± 0.05	0.61 ± 0.01	0.64 ± 0.04	0.73 ± 0.02	0.62 ± 0.02
Harvest OD_600nm_ (A.U.)	4.67 ± 0.16	10.52 ± 0.60	7.30 ± 0.10	6.79 ± 0.10	5.23 ± 0.04	8.36 ± 0.06	9.88 ± 0.17
Activity^b^ (U L_culture_^−1^)	441 ± 112	353 ± 58	24 ± 4	6 ± 0.2	36 ± 6	210 ± 93	980 ± 24
Activity^b^ (U g_protien_^−1^)	499 ± 107	222 ± 30	58 ± 5	27 ± 3	58 ± 14	247 ± 107	627 ± 220

^a^Medium recipes are found in Table S5

^b^Activity unit (U) measured for 1 mM 4-NPA at room temperature in 100 mM potassium phosphate buffer (pH 7). Activity is normalized to culture volume or mass of total protein as determined using a BCA

### Production of IsPETase in a bioreactor

Production of *Is*PETase was moved to a 2-L benchtop bioreactor with a 1.5 L working volume in order to have a larger culture volume as well as take advantage of increased aeration, better mixing, and pH control compared to the shake flasks. In addition to the bioreactor, a 100 mL culture in a 500 mL baffled shake flask was run simultaneously to determine the impact of improved aeration and pH control in the bioreactor. Complex media with glycerol was selected as it had the highest production in shake flasks **(**Table 2).

**Fig. 3:**
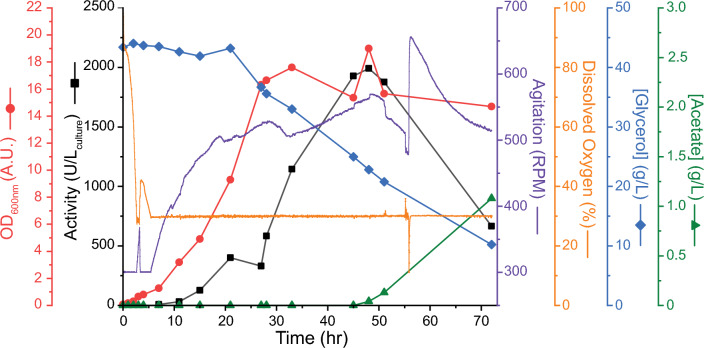
Scale-up production of *Is*PETase. *Is*PETase was produced in a 2-L bioreactor at a 1.5 L working volume scale from SHuffle T7 Express cells. Bioreactor runs used complex media with glycerol. Initial growth occurred at 37 °C followed by protein production at 20 °C after induction with 0.5 mM IPTG. Induction occurred at 3 h. Activity unit (U) were determined on 1 mM 4-NPA at room temperature in 100 mM potassium phosphate buffer (pH 7) and is normalized to culture volume (black squares). Also measured was optical density at 600 nm (red circles), agitation (purple), and dissolved oxygen (orange). Cascade control of dissolved oxygen was utilized for both reactor runs. pH was controlled between 6.8 and 7.2. Glycerol substrate (blue diamonds) and acetate product (green triangle) concentrations were measured by HPLC

Culturing in the controlled bioreactor yielded further improvements in PETase production (Fig. 3). The optical density of cultures grown in this medium reached 19 A.U. at 48 h of total culture time. The culture in the 500 mL shake flask grew similarly to that in the bioreactor, reaching a maximum cell density of 16.7 A.U. (Fig. S8a). Peak activity in the bioreactor occurred at 45 h post induction, reaching a value of 1992 units per L. Similarly, the scaled-up shake flask reached 1749 units per L culture at 45 h post induction (Fig. S8a). Enzyme activity dropped significantly over the next 24 h of culturing, with approximately 66% lost for the bioreactor culture (Fig. 3). An even more precipitous loss was found for the culture in the shake flask culture, where the difference of 2 h between sample times resulted in 94% loss (Fig. S8a).

To determine a reason for the decline in activity, glycerol consumption as well as fermentation product accumulation was tracked using HPLC. Glycerol showed steady consumption throughout the fermentation trial with increased rate as the cells reached higher cell densities. This matches the agitation and aeration rates as they were elevated to compensate for more cells in the bioreactor. Glycerol consumption continued throughout the bioreactor run and was not fully exhausted, with over 11 g L^−1^ glycerol remaining after 75-h of culturing, suggesting limitation of carbon source is not an issue. Fermentation product analysis revealed an accumulation of acetate starting at 48 h and rising throughout the remainder of the culturing time. The same was found for the shake flask sample. This suggests a metabolic imbalance at these late culture times leading to overflow metabolism of glycerol to acetate [41]. The production of acetate at 48 h seems strongly correlated with the loss of recombinant PETase activity. Since the shake flask culture did not have pH control, the accumulation of acetate and likely drop in pH might explain the large loss of active enzyme.

Excited by the increase in PETase production upon moving to the bioreactor, we sought to reproduce the data as well as purify the protein to quantify the amount produced. A repeated bioreactor run largely followed the first one, with an optical density of 13.5 and a peak activity of 2109 units per L at 43 h post induction (Fig. S8b). A time point one hour later indicated a decline in active protein amount to 1859 units per L as well as acetate accumulation to 80 mg L^−1^. At this time the entire bioreactor was harvested for purification. Using the same purification procedure as previous, we found a total of 104 ± 6 mg of PETase protein produced per L bioreactor volume, which is an almost five-fold increase from our earlier purifications using cultures grown on 2xLB, with similar protein purity (Fig. S8c). This gave us a volumetric productivity of 2.21 ± 0.13 mg L^−1^ h^−1^ for our process.

### Production and purification of PETase variants

Given the success of using SHuffle T7 Express as a protein production host, we sought to confirm if a similar benefit would be found for some of the recently evolved PETase variants. Using identical codons to the original *petase*_*Is*_, genes encoding for FAST-PETase and Hot-PETase were ordered and added to pET21b plasmid with a C-terminal hexa-histidine tag. In addition to SHuffle T7 Express, BL21(DE3) was also explored for production to see if the benefit of including disulfide bond forming machinery was beneficial for the evolved variants. Production experiments were carried out in 2xLB and shake flasks as was done for the WT IsPETase and enzyme activity was determined in soluble cell lysates.

**Fig. 4:**
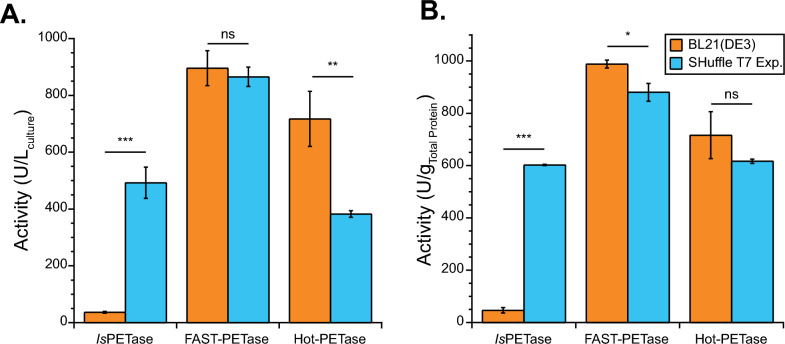
Variant PETase production in BL21(DE3) and SHuffle T7 Express. Production of PETase as measured by enzyme activity on 4-NPA in clarified cell lysates from BL21(DE3) (light blue) or SHuffle T7 Express (orange). Lysis amount was based on culture optical density. Activity was normalized to **A** culture volume and **B** total protein as measured by BCA. Activity unit (U) were measured on 1 mM 4-NPA at room temperature in 100 mM sodium phosphate buffer (pH 7.4). Average of at least 3 independent cultures shown and error bars represent the standard deviation. Shown are Student’s t-test results, ns (p > 0.05), * (p < 0.05), ** (p < 0.005), *** (p < 0.0005)

Surprisingly, PETase variants did not demonstrate the same advantage of SHuffle T7 Express, with Hot-PETase showing improved production in BL21(DE3) (Fig. 4a). As before, SHuffle T7 Express produced a statistically significant amount more *Is*PETase than BL21(DE3) (p = 1.38 × 10^–4^). The highest observed activities were for FAST-PETase in both BL21(DE3) and SHuffle T7 Express, with activities reaching 896 and 866 units per liter of culture, respectively. The lack of statistical significance between these values (p = 0.49) indicates that the disulfide bond forming machinery in SHuffle T7 Express cells has no observable impact on the expression of FAST PETase. Further, Hot-PETase was expressed significantly less effectively (p = 0.004) in SHuffle T7 Express cells than in BL21(DE3) cells, with 717 and 382 units per liter of culture, respectively. This is surprising because Hot-PETase has seven cysteine residues rather than four for *Is*PETase. Similar trends in production amount were found when normalizing activity in lysate to total protein amount (Fig. 4b).

Enzyme purification followed by characterization was carried out to determine the amount of PETase variants produced as well as ensure high enzyme activity. Production experiments were carried out in both BL21(DE3) and SHuffle T7 express to validate the activity differences seen in lysates (Table 3). FAST-PETase and Hot-PETase were better produced and purified in BL21(DE3) than SHuffle T7 Express, reaching titers of 66 mg per L and 78 mg per L, respectively. Analysis of protein purity, revealed that all proteins were purified to greater than 90% purity (Fig. S9). A reported titer of 12 mg per L was found for Hot-PETase when produced in SHuffle T7 Express [14], similar to the 19 mg per L here. Using BL21(DE3), we found a 6.5-fold improvement over what was previously observed. Another study found the amount of Hot-PETase to be roughly 110 mg per L when made in Origami 2 cells [7], a bit higher than what we achieved. Far UV CD analysis revealed that purified FAST-PETase and Hot-PETase were similarly well folded regardless of whether they were produced in BL21(DE3) or SHuffle T7 Express (Fig. S10).

**Table 3:** PETase variant purification

	Production Amount (mg/L_culture_)^a^		Purity^b^		Amount Yield^c^	
Variant	BL21(DE3)	SHuffle T7 Express	BL21(DE3)	SHuffle T7 Express	BL21(DE3)	SHuffle T7 Express
FAST	65.6 ± 0.5	31.9 ± 0.4	0.95	0.97	0.89 ± 0.07	0.40 ± 0.04
Hot	77.6 ± 1.7	19.3 ± 1.1	0.96	0.91	0.73 ± 0.09	0.29 ± 0.08

^a^Amount of PETase after purification from 0.5 L of culture. Amount was measured by BCA and comparison to BSA standard. Error represents standard deviation of two or three replicate assay samples. Resulting amount was scaled to a 1 L culture volume

^b^Purity is estimated from gel analysis of Fig. S9

^c^Amount yield represents the retention of amount in from lysate to final purified protein. Error is propagated

### Characterization of PETase variants

Specific activity was determined on 4-NPA as was done for the *Is*PETase, revealing that FAST-PETase had slightly higher activity at 25 °C compared to the other variants (Table S6, Fig. S11a). This would explain the elevated activity of the FAST-PETase compared to the other variants in lysate activity analysis, while similar purified protein amounts were found. Likewise, FAST-PETase had an increased *k*_*cat*_ compared to the other variants on 4-NPA at 25 °C (Table S6, Fig. S11b). However, when looking at catalytic efficiency, *k*_*cat*_/*K*_*M*_, on 4-NPA, all the variants were statistically similar to the *Is*PETase, suggesting the improvement of the variants is only relevant at higher temperatures and on PET substrates.

**Fig. 5:**
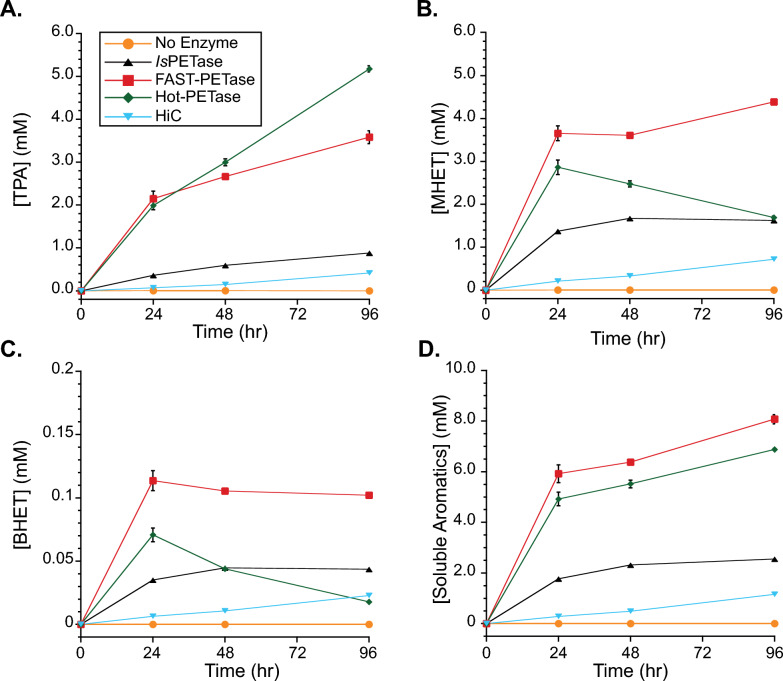
Variant PETase activity on PET microparticles. PETase activity was measured by accumulation of soluble aromatic products over time: **A** TPA, **B** MHET, **C** BHET, and **D** total soluble aromatics. Individual series in each plot represent PETase variants: *Is*PETase (black triangles, n = 1), FAST-PETase (red squares, n = 2), Hot-PETase (green diamonds, n = 2), or no enzyme (orange circles, n = 1). Also tested was HiC (light blue triangles, n = 1). All samples loaded at 1.0 mg of enzyme per gram of PET (mg g^−1^). Reactions occurred at 37 °C in 50 mM sodium phosphate buffer (pH 9), contained 30 mg PET per mL reaction, and were mixed at 1000 rpm using an Eppendorf thermomixer. Error bars represent the standard deviation of samples

Finally, activity on PET microparticles was assessed by measuring soluble aromatic product generation over time at 37 °C for all the variants in this study (Fig. 5). Additionally, the commercially-available cutinase from *Humicola isolens*, HiC, was included in the study. Analysis of total released soluble analytes after 24 h revealed that FAST-PETase liberated over 6 mM products, the most amongst any enzyme type tested in this study. Comparatively, Hot-PETase, WT PETase, HiC, and the no enzyme control liberated 5 mM, 1.8 mM, 0.28 mM, and 2 μM products, respectively at 24 h. Other works have found HiC to have low activity on PET relative to other enzymes [33]. Reacting on real PET substrates revealed the benefit of the evolved variants even at a modest reaction temperature of 37 °C. Converting all soluble products to BHET equivalents, the FAST-PETase percent conversion to soluble products was 6.84 ± 0.16%, similar to the approximately 8% from another comparative PETase study [8], albeit at different reaction conditions. From the same study, we would expect better performance for Hot-PETase at elevated temperatures.

## Discussion

We have found that *Is*PETase is optimally produced in a disulfide bond promoting host which is capable of rearrangement of improperly formed disulfide bonds due to inclusion of a cytoplasmic copy of *dsbC*. In contrast, evolved PETase variants FAST-PETase and Hot-PETase were better produced in BL21(DE3) with a reducing cytoplasm, even though FAST-PETase has the same two disulfides and Hot-PETase a third as well as a free cysteine. Despite the promise of SHuffle for making proteins that require complex disulfide bonding patterns for activity, we have found some limitations working with this host, specifically around genetic construction and purification yield. Medium screening and moving cultures to a controlled and well-mixed bioreactor further elevated the production of *Is*PETase to overcome these limitations. Accumulation of acetate in our bioreactor trials seems to be correlated with recombinant enzyme activity loss, suggesting careful monitoring of cultures to ensure titer is maximized.

Comparing our bioreactor results to produce *Is*PETase with those for *K. pastoris*, although we purified a lower protein amount per reactor volume, our strategy resulted in better per mass productivities. For our bioreactor trial with complex medium with glycerol, a CDW of 5.02 g per liter was achieved (2.69 g of CDW per L culture per OD_600_), with *Is*PETase representing 2.1% of total cell mass. Comparing to *K. pastoris* cultures, CDW was approximately 270 g per L with 1.2 g PETase per L produced [20], or just 0.4% of the total cell mass. Further, converting to a per cell mass productivity, PETase produced in SHuffle was 0.45 mg_PETase_ g_CDW_^−1^ h^−1^, while it was 0.037 mg_PETase_ g_CDW_^−1^ h^−1^ when made in *K. pastoris*. While the substrates are different for *E. coli* (glucose and glycerol) and *K. pastoris* (methanol), yield and per mass productivities are important for economic considerations as they incorporate the cost to produce biomass as well as reactor size. If a similar per mass yield was kept for SHuffle, the optical density would have to approach 130 to produce 1 g per liter, which may be possible after further fermentation process optimization (i.e. medium selection, induction strategy, substrate and nutrient feeding, dissolved oxygen level, and mixing) [27]. An advantage of *K. pastoris* is its ability to secrete enzyme which should streamline downstream processing. Lastly, glycosylation using *K. pastoris* may be both advantageous and detrimental to PETase performance. While stability is increased due to glycosylation, depending on the location of glycosylation, the active PET-binding site could be blocked, lowering the specific activity. Both increased stability and loss of activity were found for *Is*PETase made in *K. pastoris* due to glycosylation [20].

An unexpected finding of this work is that the evolved variants FAST-PETase and Hot-PETase were better produced and purified from BL21(DE3) than SHuffle T7 Express. It is known that both variants have a higher T_m_ than *Is*PETase, due to more interactions in their folded structure, especially the extra disulfide bond in Hot-PETase. It is possible that these stabilized enzymes do not require disulfides to achieve the correct folding state, keeping them from forming inclusion bodies and permitting disulfide bond formation after cell lysis. This is most likely for FAST-PETase as its disulfides are formed between consecutive cysteines, with the same being true for *Is*PETase. For FAST- and Hot-PETase it is unknown if the extra disulfide bond in PETase (C203 and C239) compared to other cutinases is necessary for activity as is true for *Is*PETase [23]. Hot-PETase has an extra disulfide bond, rationally added between C233 and C282, as well as one free cysteine, C242. Of any of the variants, this enzyme would be expected to benefit most from a disulfide bond promoting host as well as disulfide bond isomerase to properly form as the two disulfides since they are relatively close to one another in the primary sequence and they are not formed between consecutive cysteines.

Hot-PETase production as measured after purification was much lower from SHuffle T7 Express (19 mg per liter culture) compared to BL21(DE3) (77 mg per liter). Another study found that Hot-PETase production reached 110 mg per liter using Origami 2 [7], which has an oxidizing cytoplasm but lacks the DsbC chaperone. While they used different media and expression conditions, this potentially shows the benefit of disulfide bond formation and negative impact of DsbC. A similar finding was found for luciferase, which has 10 cysteines, where less active enzyme was produced in cells with an oxidizing cytoplasm and cytoplasmic DsbC compared to cells with just an oxidizing cytoplasm [24]. The authors suggest oxidized DsbC could impact the folding of a reduced protein. Alternatively, since the extra disulfide bond in Hot-PETase was not evolutionarily derived, it is possible that that the disulfide bond isomerase does not correctly recognize the designed fold to chaperone the protein to the correct confirmation, leading to misfolding and the drop off in production observed for SHuffle T7 Express.

In our work we found that purification of *Is*PETase during the IMAC step resulted in a low yield. Active protein was not found in the flow through due to exceeding the binding capacity of the column. For the bioreactor trial and scaled-up purification, we found a similar yield for *Is*PETase to the small-scale expression trials (~ 30%). Likewise, FAST-PETase and Hot-PETase had low yields for purification of 40% and 29%, respectively, when produced in SHuffle T7 Express (Table 3). The yield was much higher for proteins produced in BL21(DE3), 89% for FAST-PETase and 72% for Hot-PETase, which is more in line with expectations for the purifications performed. This suggests something in the SHuffle lysate is causing a negative impact on the purification process. Possibly, as the PETase is interacting with the IMAC resin, it is changing structure causing interaction with the overexpressed DsbC. Alternatively, the DsbC could become oxidized and cause unintended reduction of the oxidized PETase product [24]. A similar yield of between 18 and 51% was observed for production and IMAC purification of a chaperone from SHuffle [27], possibly a wide spread problem using SHuffle as a host.

Since glycerol is inexpensive and less likely to suffer from overflow metabolism into acetate compared with glucose [42, 43], its utilization as a carbon source has more advantages than simply bypassing catabolite repression [44]. At low cell densities, acetate formation does not seem to occur in ours or other studies [41] when glycerol is used as a carbon source. However, we do see acetate accumulation starting approximately 45 h after bioreactor inoculation, ultimately reaching 1 g L^−1^ (Fig. 3). Acetate accumulation is known to be toxic to cells, even at concentrations of 0.5 g L^−1^ [42], resulting in decrease biomass and recombinant protein production [43] as well as prompting reassimilation [45]. Acetate is hypothesized to have a negative impact on growth due to energetics to maintain intracellular pH or inhibition of methionine synthesis which impacts protein translation [46]. Acetate has also been proposed to have a negative effect on the stability of intracellular proteins [43] and alter transcription [47]; however, it is unknown how general the transcription changes are. We see a dramatic loss of *Is*PETase activity, even at low acetate concentrations of 0.05 g L^−1^, with over 90% loss of activity in just 3 h for a shake flask culture with uncontrolled pH. It is unknown whether acetate buildup is the cause of the loss of active PETase or if acetate accumulation happens simultaneously with the loss of recombinant protein activity. Co-factor imbalance or metabolite flux limitation likely cause the shift in glycerol metabolism to acetate at these later cell culture times. It is somewhat surprising to see such a precipitous loss of enzyme activity unless it is being scavenged for resource utilization, the enzyme has a short half-life and expression becomes limiting, or it is unfolding and moving to inclusion bodies. Regardless, our results suggest close monitoring of protein activity is necessary to catch a tight window of high production.

Our work focused on the expression and purification of soluble and active PETase. It is well established that overexpressed proteins often end up in the inclusion bodies of *E. coli* [48]. It is possible that strategies we evaluated such as higher temperature expression, increased inducer concentration, and even host selection led to increased cytoplasmic production of the enzyme into insoluble inclusion bodies. However, while purification of denatured proteins is possible, refolding to active conformations can be a challenge, especially for proteins with complex disulfide patterns [48]. This has led us to focus efforts only on soluble production in this work.

This study focused on the development of expression conditions and scale-up for *Is*PETase as well as production of recently evolved variants. Since the discovery of *Is*PETase in 2016, considerable work has gone into protein engineering this enzyme for higher stability and activity at elevated temperatures [3]. Based on the Arrhenius relationship, reaction rate should exponentially increase as the temperature is elevated. Additionally, it has been argued that as reaction temperatures approach the glass transition temperature, 60 ºC to 70 ºC for PET, catalysis is enhanced [7]. It has been established that PETase has higher activity on amorphous PET than crystalline PET [6]. However, PET re-crystallization also occurs near the glass transition temperature which is counterproductive to enzymatic breakdown [8]. From a cost standpoint, increasing temperature of reactions is not desired, rather catalysis near ambient conditions is preferred. This is especially true for remediation of plastics in the environment where temperature cannot be controlled [49]. In our work, we have found that FAST-PETase and Hot-PETase outperformed *Is*PETase based on soluble aromatics formed from PET microparticles at 37 ºC, but reaction rates on 4-NPA were nearly the same at 25 ºC. *Is*PETase has been shown to be quite active at low temperatures, producing a high degree of ester bonds hydrolyzed in just minutes [50]. At lower temperatures, the advantages of the evolved PETases might not be as extreme as at higher temperatures where *Is*PETase, or even FAST-PETase are not stable [8].

## Conclusions

Through this work we have shown that wild-type *Is*PETase is better produced in the cytoplasm of *E. coli* using a disulfide bond forming host with disulfide bond isomerase. Production was increased by 13-fold over often used BL21(DE3) and purity was enhanced due to overexpression. *Is*PETase was purified from SHuffle T7 Express as a soluble, well-folded protein with activity on PET microparticles that is on par with other studies. Moving to another complex medium that was supplemented with glycerol and to a controlled bioreactor, protein production reached 104 mg per liter after 46 h of culturing, with per cell mass productivities greater than what has been achieved with *K. pastoris*. The production benefit of SHuffle T7 Express was not found for evolved FAST-PETase and Hot-PETase, which were better made and purified from BL21(DE3). These results are expected to benefit researchers who need to produce significant quantities of *Is*PETase and related variants in a lab environment for process engineering studies to degrade PET plastics at large scale.

## Supplementary Information

Supplementary Material 1.

Supplementary Material 2.

## Data Availability

Data is provided within the manuscript or supplementary information files.

## Abbreviations

4-NPA: 4-Nitrophenyl acetate
BCA: Bicinchoninic acid assay
BHET: Bis(2-hydroxyethyl) terephthalic acid
BSA: Bovine serum albumin
CDW: Cell dry weight
FPLC: Fast-protein liquid chromatography
HiC: Cutinase from *Humicola insolens*
HPLC: High-performance liquid chromatography
IMAC: Immobilized metal affinity chromatography
IPTG: Isopropyl ß-D-1-thiogalactopyranoside
LB: Luria Bertani
MHET: 2-Hydroxyethyl terephthalic acid
OD_600_: Optical density measured at 600 nm
PET: Polyethylene terephthalate
RCF: Relative centrifugal force
RNAP: RNA polymerase
RPM: Revolutions per minute
SDS-PAGE: Sodium dodecyl sulfate polyacrylamide gel electrophoresis
TB: Terrific broth
TPA: Terephthalic acid
U: Unit of enzyme activity
UV: Ultraviolet
v/v: Volume per volume
w/v: Weight per volume
YT: Yeast tryptone
