# Supplementary material for: Increased cytoplasmic expression of PETase enzymes in E. coli
Source: Microb Cell Fact. 2024 Nov 25;23:319. doi: 10.1186/s12934-024-02585-w (PMC11587651; doi:10.1186/s12934-024-02585-w)
Supplement: Supplementary file 1 — Supplementary Material 1. [file 12934_2024_2585_MOESM1_ESM.pdf]

Supplementary Information for Increased cytoplasmic expression of PETase enzymes in *E. coli*

Luke M. Carter, Chris E. MacFarlane, Samuel P. Karlock, Tridwip Sen, Joel L. Kaar, Jason A. Berberich, Jason T. Boock

Supplementary Note 1

We attempted to further increase *Is*PETase production by utilizing a different plasmid, pET24a, as well as co-expressing with chaperones. The pET24a plasmid is similar to pET21b, but has a kanamycin marker instead of amplicillin. *Is*PETase production using the pET24a plasmid was comparable to expression from pET21b (**Table S2**). The production from pET24a was found to be 309 units per liter of culture, which was lower than that for pET21, and 1146 units per gram total protein, which was higher than that for pET21b but not statistically significant (p=0.27). The optical densities for the pET24a were typically 2.9 ± 0.1, lower than that for pET21b. Due to the production per volume for pET24a being lower than that for pET21b, we decided to use pET21b for the remainder of this work.

Others have shown that co-expression of the chaperone GroEL/ES to promote increased soluble production of a PETase mutant [1]. We paired the same pGro7 plasmid, which encodes *groEL/ES* under the control of an arabinose-inducible promoter, with our pET21b *Is*PETase in SHuffle T7 Express finding a total activity of 309 units per liter culture when samples were induced with both arabinose and IPTG, which was slightly lower than the 319 units per liter culture for samples without pGro7 produced on the same day, although not statistically significant (p=0.48) (**Table S2**). This suggests that inclusion of a chaperone does not impact *Is*PETase production, contrary to what was seen for a PETase mutant [1]; however, details about inducer concentration were not included in the previous work and an autoinduction medium was used for production potentially limiting comparison.

Table S1: *Is*PETase production in BL21(DE3)

Plasmid	[IPTG] (mM)	Production Time[a] (hr)	Induction Temperature (°C)	Activity[b] ($U\ L_{culture}^{-1}$)
pET21b *Is*PETase	0.5	24	20	14.5
pET21b *Is*PETase	0.05	24	20	14.7
pET21b *Is*PETase	0.5	48	20	14.5
pET21b *Is*PETase	0.5	24	37	2.4
pET21b Empty	0.5	24	20	2.6

[a] Time between induction and harvest.
[b] Activity unit (U) measured on 1 mM 4-NPA at room temperature in 100 mM potassium phosphate buffer (pH 7). Activity unit is normalized to 1 L of culture volume.

Table S2: Comparison of lysis methods

	Total Protein ($mg\ L_{culture}^{-1}$)		Activity[a] ($U\ L_{culture}^{-1}$)		Activity[a] ($U\ g_{TotalProtein}^{-1}$)	
Strain	Volume[b]	OD_{600}[c]	Volume[b]	OD_{600}[c]	Volume[b]	OD_{600}[c]
BL21(DE3)	378 ± 40	792 ± 135	13 ± 1	37 ± 4	34 ± 5	47 ± 10
SHuffle	479 ± 59	817 ± 94	367 ± 58	493 ± 55	777 ± 152	603 ± 3

[a] Activity unit (U) measured on 1 mM 4-NPA at room temperature in 100 mM potassium phosphate buffer (pH 7). Activity is normalized to culture volume or mass of total protein as determined using a BCA. Average of 3 to 9 independent cultures. Reported error is standard deviation.
[b] Lysis based on culture volume. A 10 mL culture was lysed in a volume of 0.5 mL.
[c] Lysis based on cell density as measured by OD_{600}. The OD_{600} of the resuspended pellet is expected to be 100 A.U..

Table S3: Colony screening for *Is*PETase production in C41(DE3)

Colony Number	Activity[a] (U $L_{culture}^{-1}$)	Activity[a] (U $g_{TotalProtein}^{-1}$)
#1	3.1	8.4
#2	3.0	8.8
#3	3.0	8.0
#4	2.7	7.9
#5	2.6	7.3
#6	2.8	8.0
#7	3.1	7.6
#8	2.8	7.5

[a] Activity unit (U) measured on 1 mM 4-NPA at room temperature in 100 mM potassium phosphate buffer (pH 7). Activity is normalized to culture volume or mass of total protein as determined using a BCA.

Table S4: Strain, construct, plasmid and chaperone screening for *Is*PETase production

Strain	PETase Construct	PETase Plasmid	Additional Plasmid	Inducer[a]	Activity[b] (U $L_{culture}^{-1}$)	Activity[b] (U $g_{TotalProtein}^{-1}$)
SHuffle	ss*Is*PETase[c]	pET21b	-	IPTG	10 ± 4	34 ± 16
C41	ss*Is*PETase	pET21b	-	IPTG	108 ± 12	262 ± 19
				p-value[f]	*5.9x10*$^{-4}$	*2.6x10*$^{-4}$
SHuffle	*Is*PETase	pET21b	-	IPTG	378 ± 32	1000 ± 53[d]
SHuffle	*Is*PETase	pET24a	-	IPTG	310 ± 10	1146 ± 192
				p-value	*0.022*	*0.27*
SHuffle	*Is*PETase	pET21b	pGro7[e]	IPTG	291 ± 83	589 ±186
SHuffle	*Is*PETase	pET21b	pGro7	Arabinose	6 ± 1	22 ± 3
SHuffle	*Is*PETase	pET21b	pGro7	IPTG, Arabinose	309 ± 22	573 ± 34
SHuffle	*Is*PETase	pET21b	-	IPTG	319 ± 1	640 ± 28
				p-value	*0.48*	*0.058*

[a] IPTG was added to a final concentration of 0.5 mM. Arabinose was added to a final concentration of 0.5 % (w/v).
[b] Activity unit (U) measured on 1 mM 4-NPA at room temperature in 100 mM potassium phosphate buffer (pH 7). Activity is normalized to culture volume or mass of total protein. Average of 3 or 4 independent cultures. Reported error is standard deviation. C41 pET21b ss*Is*PETase is the average of 2 independent cultures.
[c] ss*Is*PETase contains a signal sequence. Plasmid was purchased from Addgene (#112202).
[d] For pET21b and pET24a comparison, a Bradford assay was used to quantify total protein content in clarified lysates through comparison to BSA standard. All other total protein quantified by BCA.
[e] pGro7 (Takara) contains *E. coli groEL/ES* under the control of an arabinose-inducible promoter.
[f] p-value calculated between samples above using two-tailed Student's t-Test.

Table S5: Medium recipes[a]

	2xLB	TB	Defined	Semi-Defined	Complex
Yeast extract	10 g	24 g	-	1 g	5 g
Tryptone	20 g	-	-	-	10 g
Casein	-	12 g	-	-	-
Additional carbon source	0 or 5 g glucose	4 mL glycerol	5 g glucose	5 g glucose	5 g glucose or 35 g glycerol
NaCl	5 g	-	-	-	10 g
KH_2PO_4	-	2.2 g	13.3 g	13 g	8.34 g
K_2HPO_4	-	9.4 g	-	10 g	6.74 g
NaH_2PO_4	-	-	-	4.6 g	-
$(NH_4)_2HPO4{\bullet}H_2O$	-	-	4 g	3 g	-
Citric acid	-	-	1.7 g	-	-
$MgSO_4{\bullet}7H_2O$	-	-	1.2 g	2 g	0.5 g
$CaCl_2{\bullet}2H_2O$	-	-	-	73.8 mg	50 mg
Thiamin HCl	-	-	4.5 mg	-	-
Metals mix	-	-	1x	1x	1x
Source			[2]	[3]	[4]

[a] All quantities provided for 1 L of media.
[b] Trace metals mix (final concentration per L): 0.1 mg H_3BO_3, 0.1 mg $CoCl_2{\bullet}6H_2O$, 25 mg $ZnSO_4{\bullet}7H_2O$, 4 mg $MnCl_2{\bullet}4H_2O$, 0.1 mg $Na_2MoO_4{\bullet}2H_2O$, 1.8 mg $CuSO_4{\bullet}5H_2O$, 20 mg $FeSO_4{\bullet}7H_2O$, 0.1 mg $NiSO_4{\bullet}6H_2O$. A 100x metals mix was diluted to 1x for each medium.

Table S6: Kinetic parameters

	Specific Activity[a]		Kinetic Parameters[b]		
Variant[c]	U mg^{-1}	U nmol^{-1}	K_m (mM)	k_{cat} (s^{-1})	k_{cat}/K_M (s^{-1} mM^{-1})
*Is*PETase	5.26 ± 0.12	183 ± 4	8.16 ± 0.67	30.3 ± 1.1	3.71 ± 0.34
FAST	7.49 ± 0.22	261 ± 8	11.33 ± 1.11	45.7 ± 2.3	4.03 ± 0.44
Hot	4.20 ± 0.08	146 ± 3	9.61 ± 0.91	33.0 ± 1.5	3.43 ± 0.36

[a] Reactions contained 1 mM 4-NPA per well in 100mM potassium phosphate buffer (pH 7) and were carried out at 25°C. U is $\mu mol_{4\text{-NPA}}$ consumed per minute with blank reaction without PETase subtracted. Data shown in Supplemental Figure S3 and S11.
[b] Kinetic parameters for 4-NPA. Reactions were carried out in 100mM potassium phosphate buffer (pH 7) at 25°C. Initial rate of conversion of 4-NPA was measured. Blank reaction rate with substrate only was subtracted. Data shown in Supplemental Figure S4 and S11.
[c] All PETase variants were purified from SHuffle T7 Express.

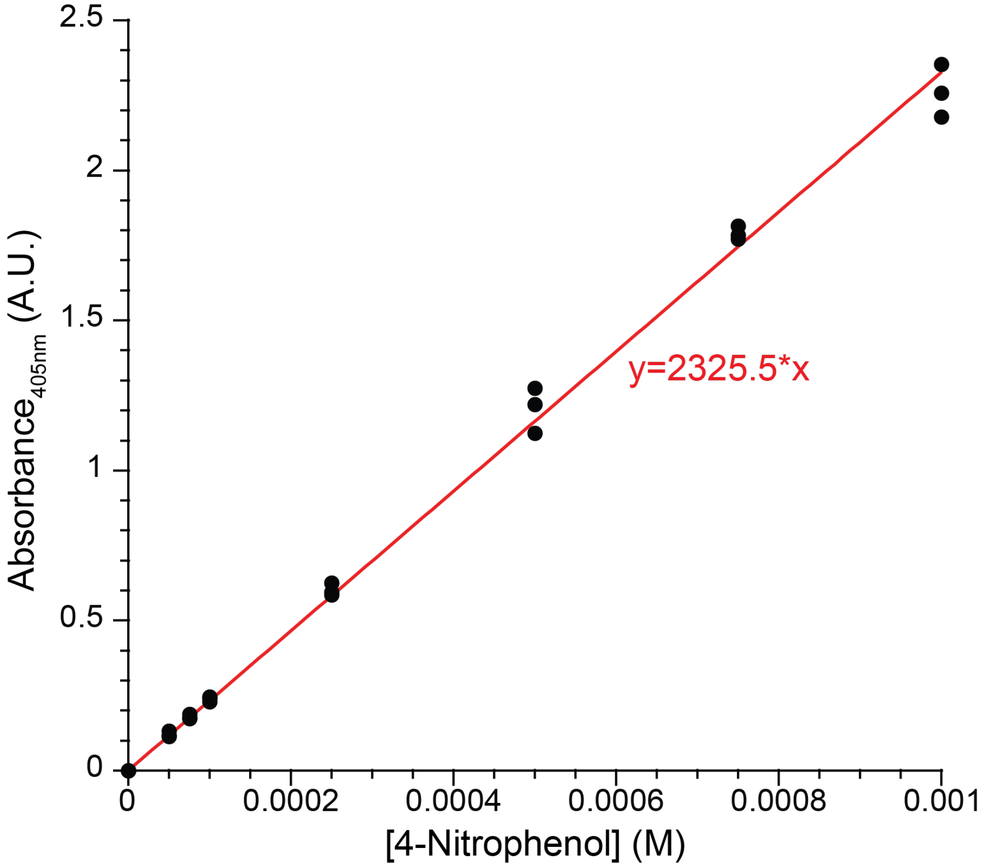

Figure S1: 4 nitrophenol standard curve. Absorbance at 405 nm as a function of 4 nitrophenol concentration. 4 nitrophenol is dissolved in 100 mM potassium phosphate (pH 7.0). Measurements made in 100 μL volume in a clear 96-well plate using a plate reader. The pathlength is assumed to be 0.2 cm. Each concentration was measured in triplicate.

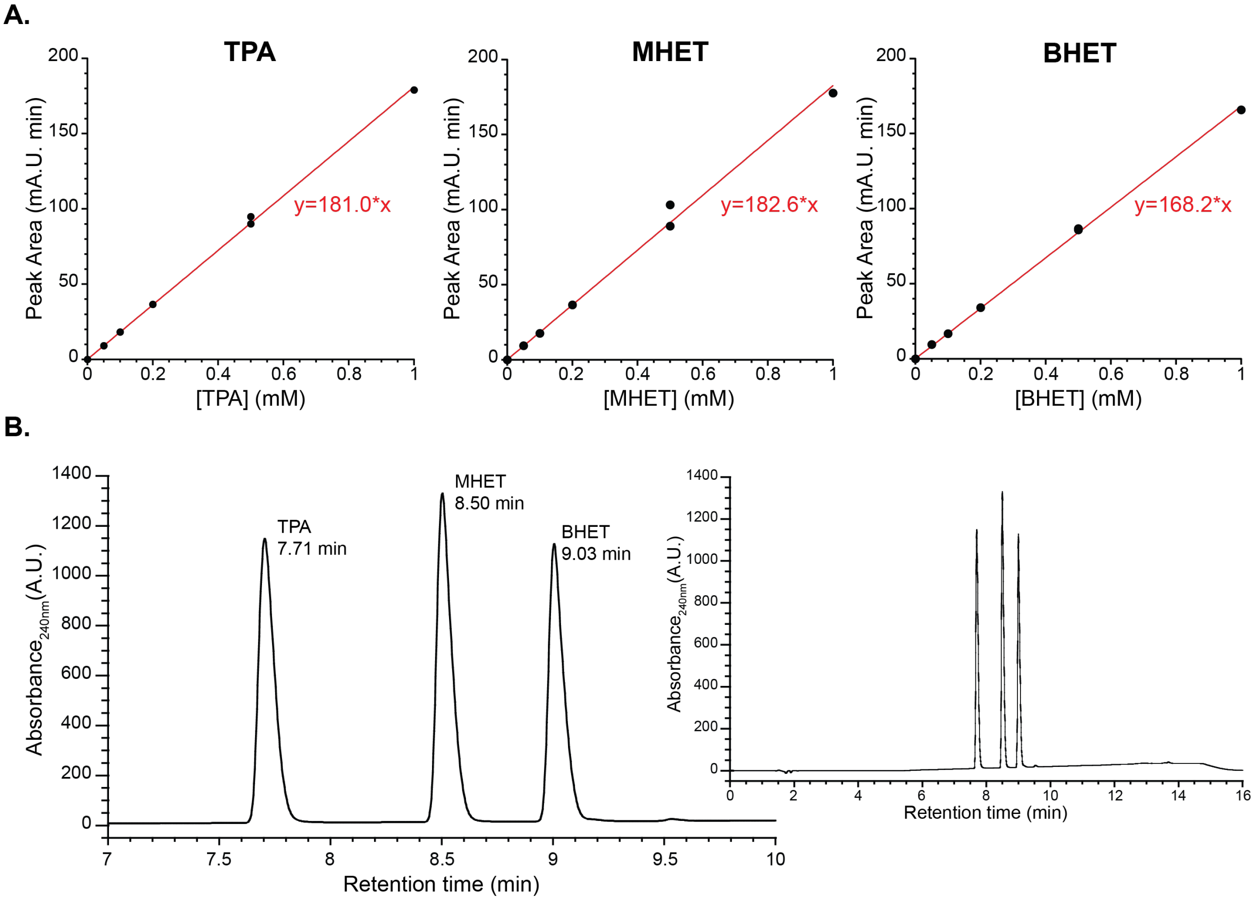

Figure S2: Soluble aromatic product standard curve and HPLC trace. A) Peak area as a function of analyte concentration for TPA, MHET and BHET. TPA was dissolved in 20 mM sodium phosphate (pH 7.4), which was returned to pH 7 upon dissolving TPA using NaOH. BHET was dissolved in 24 % (v/v) acetonitrile, 76 % 100 mM potassium phosphate (pH 7). MHET was synthesized from BHET using HiC enzyme as described in the methods. B) HPLC trace for as samples with 0.5 mM TPA, 0.5 mM MHET, and 0.5 mM BHET. Absorbance was measured at 240 nm. Peak identity and retention times were determined via HPLC runs with a single compound. Inset shows HPLC chromatogram for the entire run.

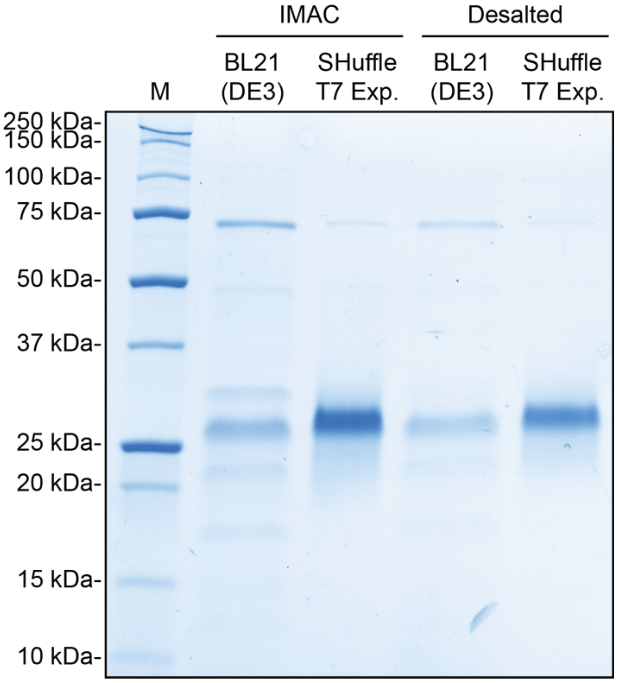

Figure S3: SDS-PAGE of *Is*PETase purification. PETase was purified from BL21(DE3) and SHuffle T7 Express strains using IMAC followed by desalting into 30 mM sodium phosphate (pH 7.4), 50 mM sodium chloride. Lanes were loaded with 2.5 µg of total protein as determined by BCA. Any kDa gel were used for separation followed by Stain-free activation. Analysis of the gel was performed using Image Lab.

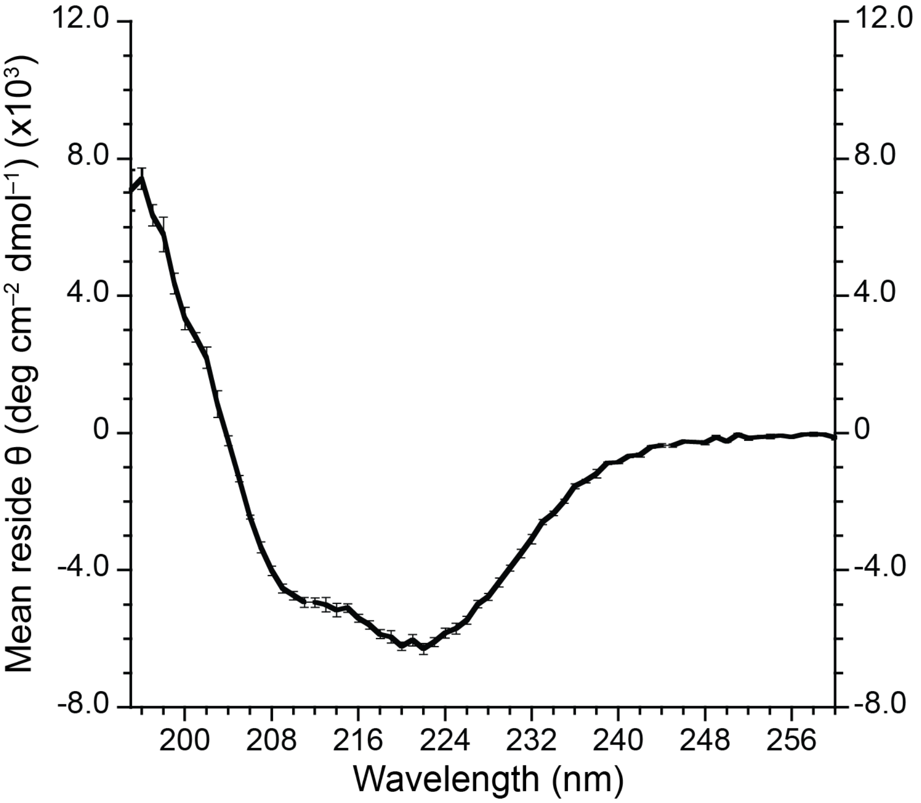

Figure S4: Circular dichroism of purified *Is*PETase. Far UV CD spectrum of *Is*PETase purified from SHuffle T7 Express. Mean residue ellipticity was determined using 0.03 mg/mL *Is*PETase in 10 mM sodium borate (pH 8) buffer. A 10 mm pathlength quartz cuvette was used at 25ºC. Show is the average of three scans of one sample and error bars represent the standard deviation.

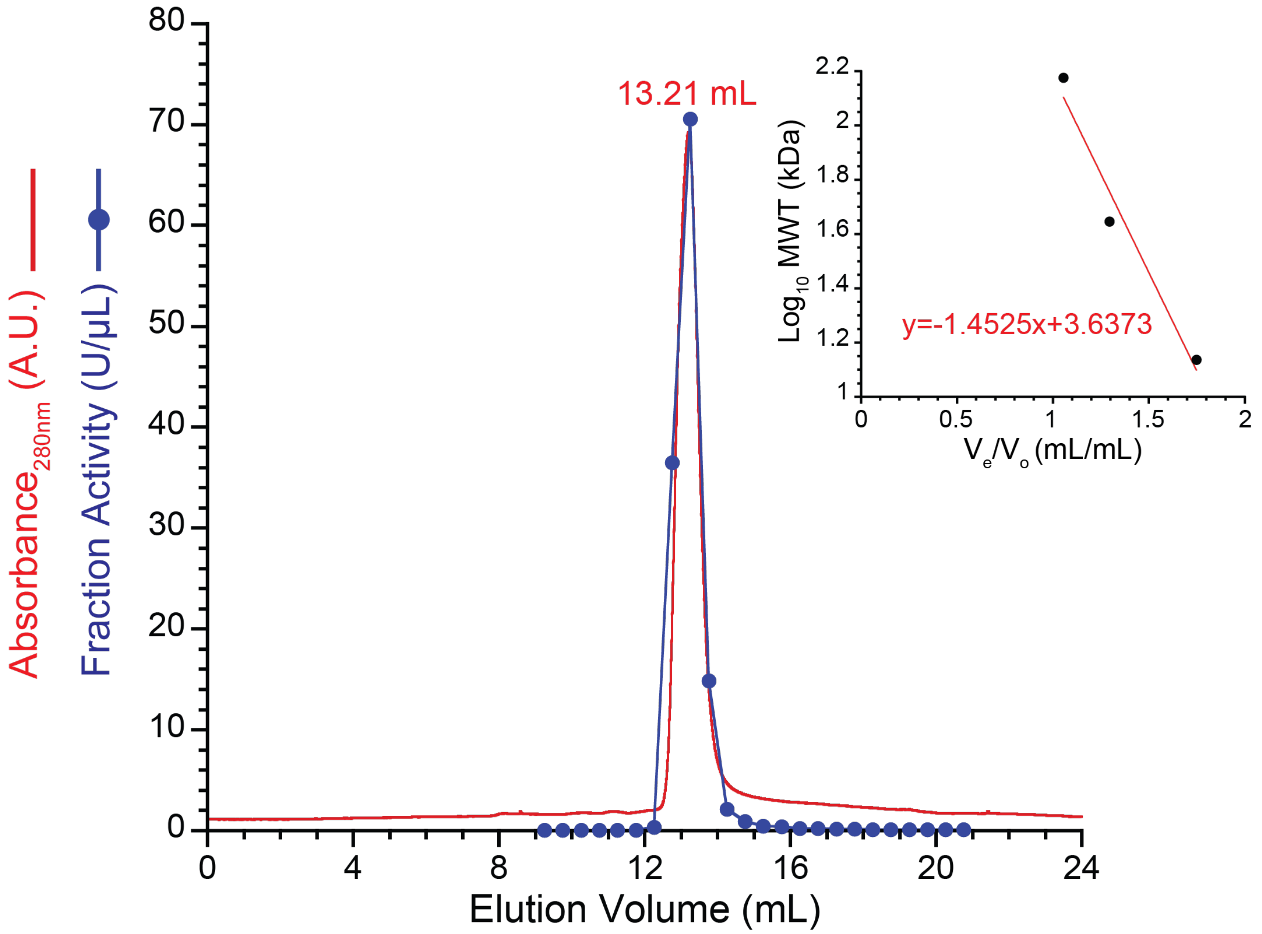

Figure S5: Size exclusion chromatography on purified *Is*PETase. Size exclusion chromatography was performed on *Is*PETase purified from SHuffle T7 Express using a Superdex 75 column and isocratic 50 mM sodium phosphate (pH 7), 30 mM sodium chloride buffer flowing at 1 mL/min. Shown is the resulting absorbance at 280 nm (red line) as a function of elution volume. Also shown is enzyme activity of 0.5 mL fractions measured on 1 mM 4-NPA at room temperature in 100 mM potassium phosphate buffer (pH 7). The inset shows elution volume for three proteins of known molecular weight. Elution volume (V_e) is normalized by void volume (V_o, 7.93 mL).

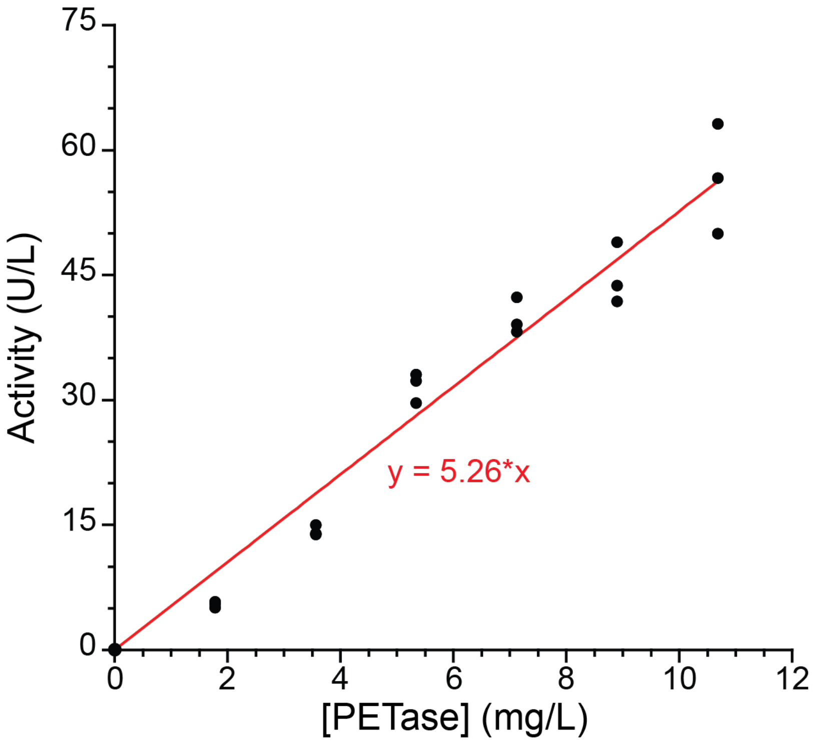

Figure S6: *Is*PETase specific activity on 4-NPA. Activity of *Is*PETase on 4-NPA as a function of purified *Is*PETase concentration. Reactions contained 1 mM 4-NPA per well in 100mM potassium phosphate buffer (pH 7) and were carried out at 25°C. U is $\mu mol_{4\text{-}NPA}$ consumed per minute with blank reaction without PETase subtracted. Linear fit was forced through the origin and is shown in red.

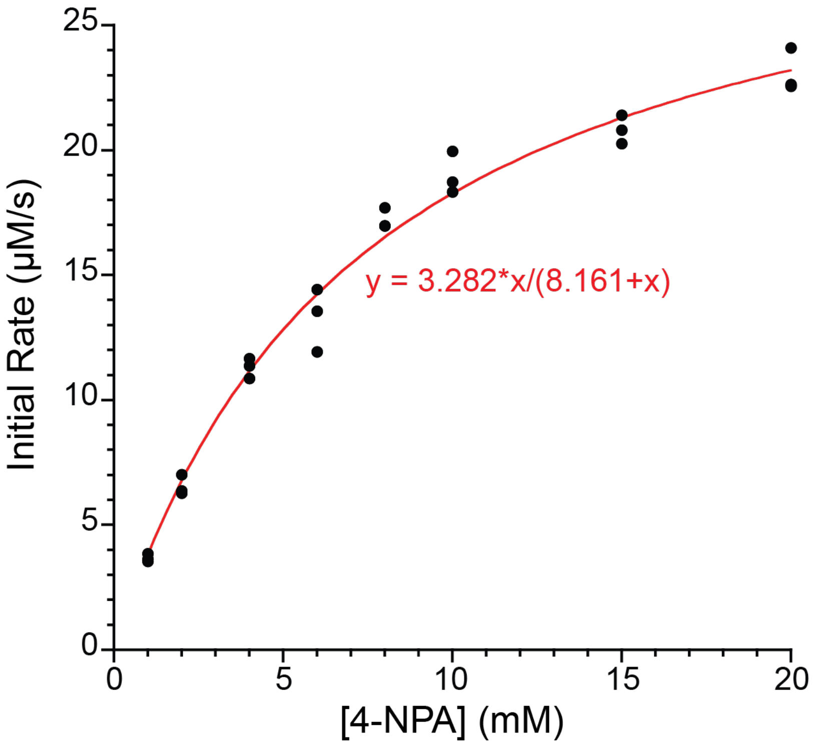

Figure S7: Michaelis-Menten plot for *Is*PETase activity on 4-NPA. Initial rates of reaction of *Is*PETase as a function of 4-NPA starting concentrations. Reactions contained 1.07 µM *Is*PETase per well in 100mM potassium phosphate buffer (pH 7) and were carried out at 25°C. Initial rate of conversion of 4-NPA was measured. Blank reaction rate with substrate only was subtracted. Fit to the Michaelis-Menten equation is shown in red.

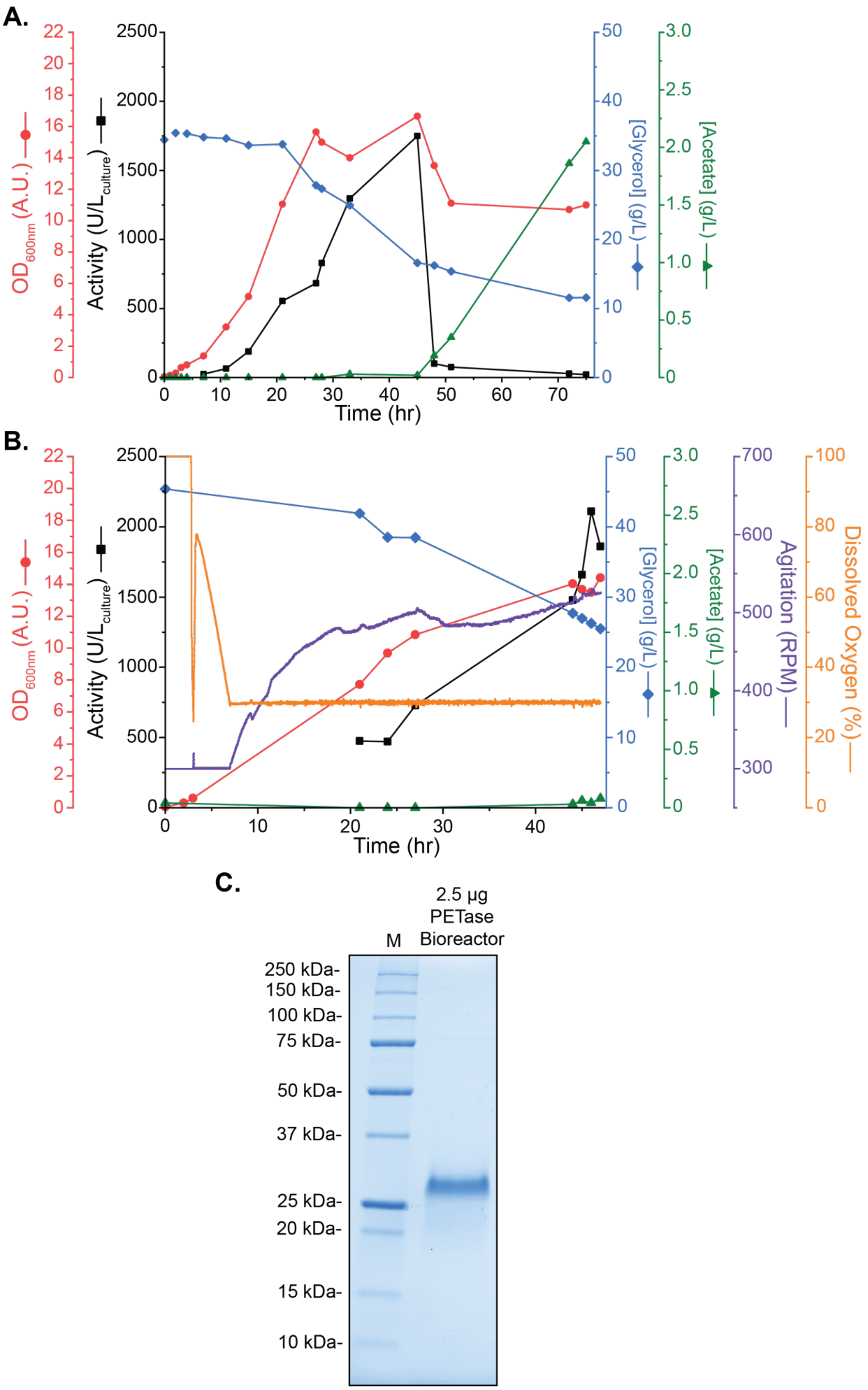

A.
OD600nm (A.U.)
Activity (U/Lculture)
[Glycerol] (g/L)
[Acetate] (g/L)
Time (hr)
B.
Agitation (RPM)
Dissolved Oxygen (%)
C.
M
2.5 µg PETase Bioreactor
250 kDa-
150 kDa-
100 kDa-
75 kDa-
50 kDa-
37 kDa-
25 kDa-
20 kDa-
15 kDa-
10 kDa-

Figure S8: *Is*PETase production using complex media with glycerol. *Is*PETase was produced in SHuffle T7 Express using complex media with glycerol in a A) 500 mL baffled shake flask with 100 mL media or B) 3-L bioreactor with 1.5 L media. Initial growth occurred at 37°C followed by protein production at 20°C after induction with 0.5 mM IPTG. Induction occurred at 2.5 hours. Enzyme activity was determined in clarified cell lysates (lysed by optical density) using 1 mM 4-NPA at room temperature in 100 mM potassium phosphate buffer (pH 7). Enzyme activity was normalized to culture volume (black squares). Also measured was optical density at 600 nm (red circles). Glycerol substrate consumption (blue diamonds) and acetate product generation (green triangles) were measured by HPLC. For the bioreactor, agitation (purple) and dissolved oxygen (orange) were measured. Cascade control of dissolved oxygen and pH control between 6.8 and 7.2 were utilized for the bioreactor trial. Bioreactor run is a replicate trial of data shown in Figure 4. C) *Is*PETase purified from the bioreactor. Cells were harvested at 47 hours total culturing time. After clarifying cell lysates, samples were purified using IMAC followed by desalting into 50 mM sodium phosphate (pH7), 30 mM NaCl. Lanes were loaded with 2.5 μg of total protein as determined by BCA. Any kDa gel (BioRad) were used for separation followed by Stain-free activation using a BioRAD imager.

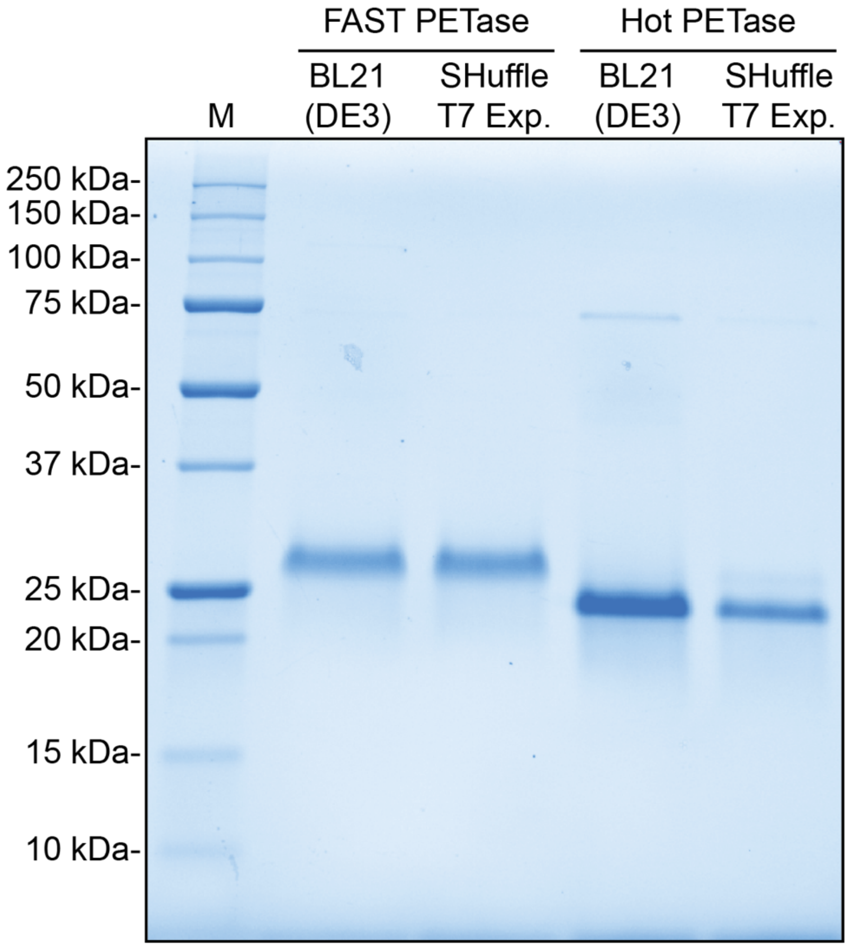

Figure S9: Purification of PETase variants. FAST-PETase or Hot-PETase was produced in BL21(DE3) or SHuffle T7 Express. After clarifying cell lysates, samples were purified using IMAC followed by desalting into 50 mM sodium phosphate (pH7), 30 mM NaCl. Lanes were loaded with 2.5 µg of total protein as determined by BCA. Any kDa gel (BioRad) were used for separation followed by Stain-free activation using a BioRAD imager.

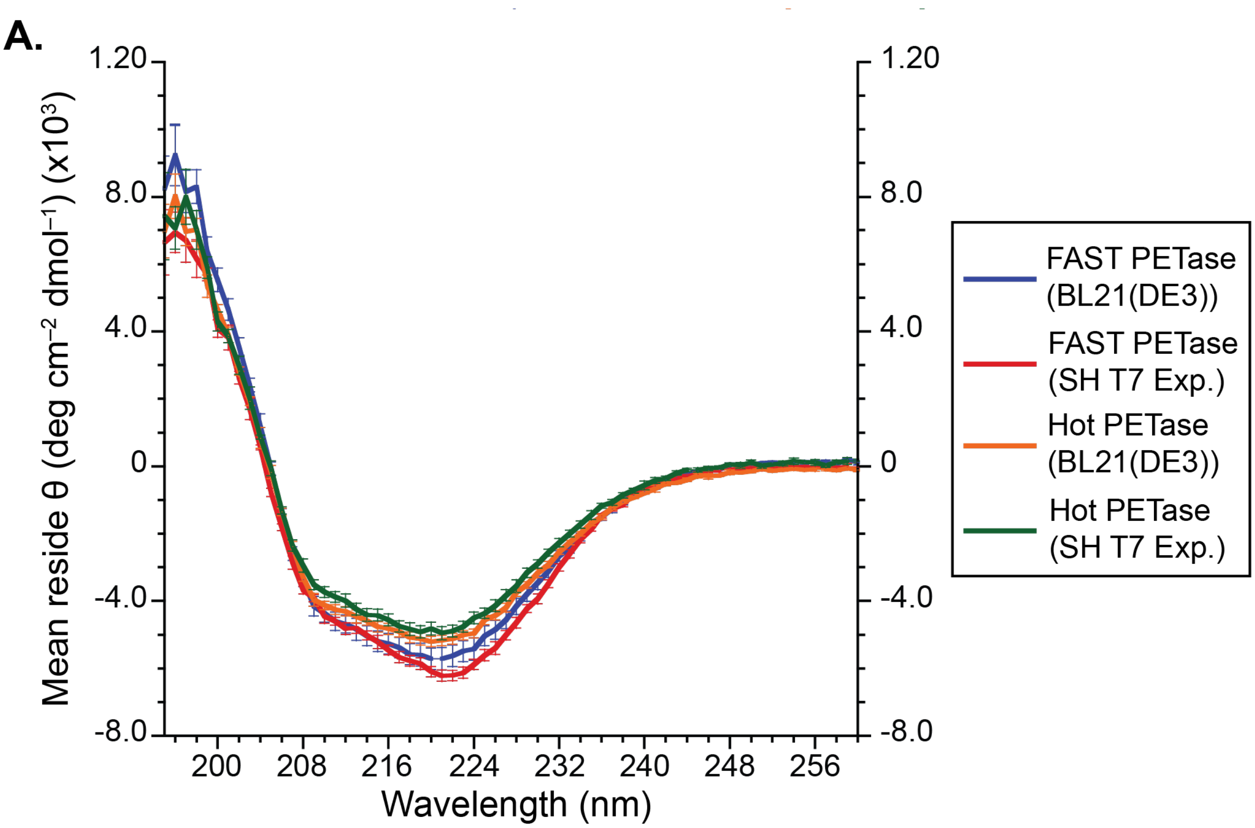

Figure S10: Circular dichroism of purified PETase variants. A) Far UV CD spectrum of FAST- or Hot-PETase purified from BL21(DE3) of SHuffle T7 Express. Mean residue ellipticity was measured using 0.03 mg/mL enzyme in 10 mM sodium borate (pH 8) buffer. A 10 mm pathlength quartz cuvette was used at 25°C. Data points are the average of three scans of one sample and error bars represent the standard deviation.

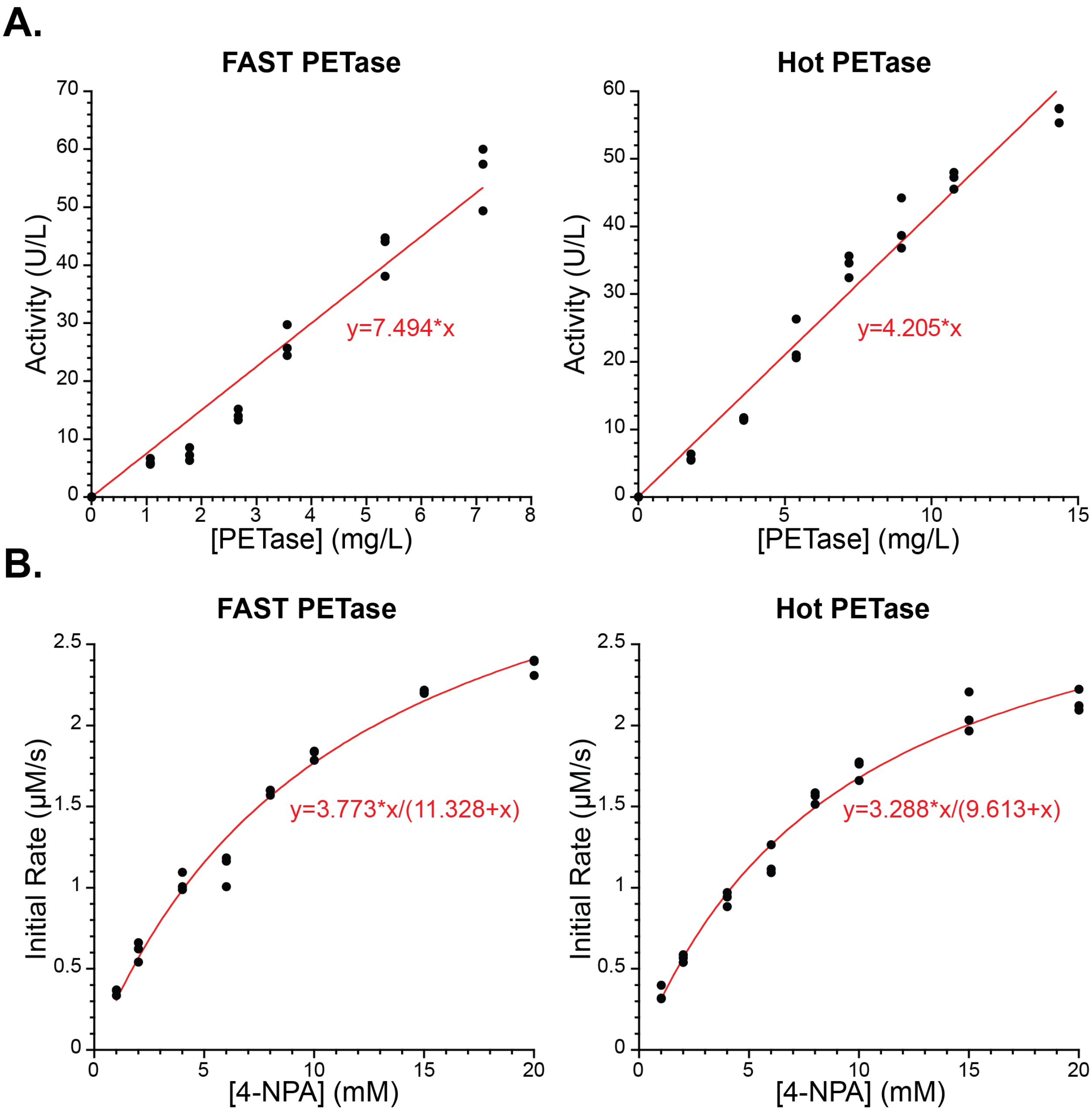

Figure S11. Characterization of PETase variants. A) Specific activity of PETase on 4-NPA as a function of purified PETase concentration. Reactions contained 1 mM 4-NPA per well in 100mM potassium phosphate buffer (pH 7) and were carried out at 25°C. U is $\mu mol_{4\text{-}NPA}$ consumed per minute with blank reaction without PETase subtracted. Linear fit is shown in red. B) Michaelis-Menten plots for PETase variants. Initial rates of reaction of PETase as a function of 4-NPA starting concentrations. Reactions contained 82.6 nM FAST PETase or 99.7 nM Hot PETase per well in 100mM potassium phosphate buffer (pH 7) and were carried out at 25°C. Initial rate of conversion of 4-NPA was measured. Blank reaction rate with substrate only was subtracted. Fit to the Michaelis-Menten equation is shown in red. For both specific activity and Michaelis-Menten measurements, PETase variants were purified from SHuffle T7 Express.

*Is*PETase Gene Sequence:

ATGCAGACTAATCCATACGCCCGCGGACCTAACCCTACGGCGGCGAGTTTGGAGGCTTCTGCTG
GTCCGTTCACAGTTCGTTCTTTTACGGTCTCGCGTCCAAGCGGGTACGGCGCTGGAACCGTCTA
CTATCCCACTAACGCTGGAGGCACAGTAGGAGCTATCGCCATTGTCCCCGGCTACACTGCTCGC
CAAAGCAGTATCAAATGGTGGGGTCCGCGCTTGGCCTCACATGGGTTTGTTGTGATTACCATCG
ACACTAATTCCACATTGGACCAGCCATCCTCACGTTCTTCACAACAAATGGCTGCCTTACGCCA
AGTGGCGTCGCTTAATGGGACTTCTAGTTCACCAATCTACGGTAAGGTAGATACTGCTCGCATG
GGAGTTATGGGATGGAGCATGGGCGGTGGGGGGAGCCTTATTTCCGCAGCGAATAACCCATCCC
TGAAGGCTGCCGCACCTCAGGCCCCATGGGATTCGTCAACTAATTTCAGCTCGGTCACCGTCCC
TACTCTTATTTTCGCTTGTGAAAACGACAGTATCGCACCTGTTAATTCATCGGCACTGCCAATC
TATGATTCCATGTCCCGTAACGCTAAGCAATTCCTGGAGATTAACGGGGGAAGTCACTCGTGCG
CTAATAGTGGGAACAGTAATCAAGCTCTTATCGGGAAGAAGGGTGTTGCATGGATGAAACGCTT
TATGGACAACGACACACGTTATAGTACCTTTGCATGCGAAAATCCTAATTCTACGCGCGTGAGT
GACTTCCGCACGGCAAATTGCAGCCTCGAGcaccaccatcaccaccactga

*Is*PETase Amino Acid Sequence:

MQTNPYARGPNPTAASLEASAGPFTVRSFTVSRPSGYGAGTVYYPTNAGGTVGAIAIVPGYTAR
QSSIKWWGPRLASHGFVVITIDTNSTLDQPSSRSSQQMAALRQVASLNGTSSSPIYGKVDTARM
GVMGWSMGGGGSLISAANNPSLKAAAPQAPWDSSTNFSSVTVPTLIFACENDSIAPVNSSALPI
YDSMSRNAKQFLEINGGSHSCANSGNSNQALIGKKGVAWMKRFMDNDTRYSTFACENPNSTRVS
DFRTANCSLEHHHHHH*

FAST-PETase Gene Sequence:

ATGCAGACTAATCCATACGCCCGCGGACCTAACCCTACGGCGGCGAGTTTGGAGGCTTCTGCTG
GTCCGTTCACAGTTCGTTCTTTTACGGTGTCGCGTCCAAGCGGGTACGGCGCTGGAACCGTCTA
CTATCCCACTAACGCTGGAGGCACAGTAGGAGCTATCGCCATTGTCCCCGGCTACACTGCTCGC
CAAAGCAGTATCAAATGGTGGGGTCCGCGCTTGGCCTCACATGGGTTTGTTGTGATTACCATCG
ACACTAATTCCACATTGGACCAGCCAgaaTCACGTTCTTCACAACAAATGGCTGCCTTACGCCA
AGTGGCGTCGCTTAATGGGACTTCTAGTTCACCAATCTACGGTAAGGTAGATACTGCTCGCATG
GGAGTTATGGGATGGAGCATGGGCGGTGGGGGGAGCCTTATTTCCGCAGCGAATAACCCATCCC
TGAAGGCTGCCGCACCTCAGGCCCCGTGGcatTCGTCAACTAATTTCAGCTCGGTCACCGTCCC
TACTCTTATTTTCGCTTGTGAAAACGACAGTATCGCACCTGTTAATTCATCGGCACTGCCAATC
TATGATTCCATGTCCcagAACGCTAAGCAATTCCTGGAGATTaaaGGGGGAAGTCACTCGTGCG
CTAATAGTGGGAACAGTAATCAAGCTCTTATCGGGAAGAAGGGTGTTGCATGGATGAAACGCTT
TATGGACAACGACACACGTTATAGTACCTTTGCGTGCGAAAATCCTAATTCTACGgcgGTGAGT
GACTTCCGCACGGCAAATTGCAGCCTCGAGcaccaccaccaccaccactga

FAST-PETase Amino Acid Sequence:

MQTNPYARGPNPTAASLEASAGPFTVRSFTVSRPSGYGAGTVYYPTNAGGTVGAIAIVPGYTAR
QSSIKWWGPRLASHGFVVITIDTNSTLDQPESRSSQQMAALRQVASLNGTSSSPIYGKVDTARM
GVMGWSMGGGGSLISAANNPSLKAAAPQAPWHSSTNFSSVTVPTLIFACENDSIAPVNSSALPI
YDSMSQNAKQFLEIKGGSHSCANSGNSNQALIGKKGVAWMKRFMDNDTRYSTFACENPNSTAVS
DFRTANCSLEHHHHHH*

Hot-PETase Gene Sequence:

ATGCAGACTAATCCATACGCCCGCGGACCTAACCCTACGGCGGCGAGTTTGGAGGCTTCTGCTG
GTCCGTTCACAGTTCGTTCTTTTACGGTCgcgCGTCCAgtgGGGTACGGCGCTGGAACCGTCTA
CTATCCCACTAACGCTGGAGGCACAGTAGGAGCTATCGCCATTGTCCCCGGCTACACTGCTacc
CAAAGCAGTATCaacTGGTGGGGTCCGCGCTTGGCCTCACATGGGTTTGTTGTGATTACCATCG
ACACTAATTCCACATTGGACaaaCCAgaaTCACGTTCTTCACAACAAATGGCTGCCTTACGCCA
AGTGGCGTCGCTTAATGGGACTTCTAGTTCACCAATCTACGGTAAGGTAGATACTGCTCGCggc
GGAGTTATGGGATGGAGCATGGGCGGTGGGGGGAGCCTTATTTCCGCAGCGAATAACCCATCCC
TGAAGGCTGCCGCAgtgatgGCCCCGTGGcatTCGTCAACTAATTTCAGCTCGGTCACCGTCCC
TACTCTTATTTTCGCTTGTGAAAACGACcgtATCGCACCTGTTaaagagtatGCACTGCCAATC
TATGATTCCATGTCCctgAACGCTAAGCAATTCCTGGAGATTtgcGGGGGAAGTCACTCGTGCG
CTtgtAGTGGGAACAGTAATCAAGCTCTTATCGGGatgAAGGGTGTTGCATGGATGAAACGCTT
TATGGACAACGACACACGTTATAGTcagTTTGCGTGCGAAAATCCTAATTCTACGgcgGTGtgt
GACTTCCGCACGGCAAATTGCAGCCTCGAGcaccaccaccaccaccactga

Hot-PETase Amino Acid Sequence:

MQTNPYARGPNPTAASLEASAGPFTVRSFTVARPVGYGAGTVYYPTNAGGTVGAIAIVPGYTAT
QSSINWWGPRLASHGFVVITIDTNSTLDKPESRSSQQMAALRQVASLNGTSSSPIYGKVDTARG
GVMGWSMGGGGSLISAANNPSLKAAAVMAPWHSSTNFSSVTVPTLIFACENDRIAPVKEYALPI
YDSMSLNAKQFLEICGGSHSCACSGNSNQALIGMKGVAWMKRFMDNDTRYSQFACENPNSTAVC
DFRTANCSLEHHHHHH*

Supplementary Information References:

1. Aer L, Jiang Q, Gul I, Qi Z, Feng J, Tang L: **Overexpression and kinetic analysis of Ideonella sakaiensis PETase for polyethylene terephthalate (PET) degradation.** *Environ Res* 2022, **212:**113472.
2. Riesenberg D, Schulz V, Knorre WA, Pohl HD, Korz D, Sanders EA, Ross A, Deckwer WD: **High cell density cultivation of Escherichia coli at controlled specific growth rate.** *J Biotechnol* 1991, **20:**17-27.
3. Studier FW: **Protein production by auto-induction in high density shaking cultures.** *Protein Expr Purif* 2005, **41:**207-234.
4. Santos J: **Cultivation of Escherichia coli BL21 (DE3) For Production of L-Asparaginase II.** University of Sao Paulo São Paulo, Brazil2017.
